# Selection of density standard and X–ray tube settings for computed digital absorptiometry in horses using the k–means clustering algorithm

**DOI:** 10.1186/s12917-025-04591-5

**Published:** 2025-03-13

**Authors:** Bernard Turek, Marek Pawlikowski, Krzysztof Jankowski, Marta Borowska, Katarzyna Skierbiszewska, Tomasz Jasiński, Małgorzata Domino

**Affiliations:** 1 Department of Large Animal Diseases and Clinic, Institute of Veterinary Medicine, Warsaw University of Life Sciences (WULS – SGGW), Nowoursynowska 100, Warsaw, 02-797 Poland; 2 Institute of Mechanics and Printing, Warsaw University of Technology, Narbutta 85, Warsaw, 02-524 Poland; 3 Institute of Biomedical Engineering, Faculty of Mechanical Engineering, Białystok University of Technology, Wiejska 45C, Bialystok, 15-351 Poland

**Keywords:** Computed digital absorptiometry, Bone mineral density, Radiological signs, Distal limbs, Horse

## Abstract

**Background:**

In veterinary medicine, conventional radiography is the first–choice method for most diagnostic imaging applications in both small animal and equine practice. One direction in its development is the integration of bone density evaluation and artificial intelligence–assisted clinical decision–making, which is expected to enhance and streamline veterinarians’ daily practices. One such decision–support method is k–means clustering, a machine learning and data mining technique that can be used clinically to classify radiographic signs into healthy or affected clusters. The study aims to investigate whether the k–means clustering algorithm can differentiate cortical and trabecular bone in both healthy and affected horse limbs. Therefore, identifying the optimal computed digital absorptiometry parameters was necessary.

**Methods and results:**

Five metal–made density standards, made of pure aluminum, aluminum alloy (duralumin), cuprum alloy, iron–nickel alloy, and iron–silicon alloy, and ten X–ray tube settings were evaluated for the radiographic imaging of equine distal limbs, including six healthy limbs and six with radiographic signs of osteoarthritis. Density standards were imaged using ten combinations of X–ray tube settings, ranging from 50 to 90 kV and 1.2 to 4.0 mAs. The relative density in Hounsfield units was firstly returned for both bone types and density standards, then compared, and finally used for clustering. In both healthy and osteoarthritis–affected limbs, the relative density of the long pastern bone (the proximal phalanx) differed between bone types, allowing the k–means clustering algorithm to successful differentiate cortical and trabecular bone.

**Conclusion:**

Density standard made of duralumin, along with the 60 kV, 4.0 mAs X–ray tube settings, yielded the highest clustering metric values and was therefore considered optimal for further research. We believe that the identified optimal computed digital absorptiometry parameters may be recommended for further researches on the relative quantification of conventional radiographs and for distal limb examination in equine veterinary practice.

**Supplementary Information:**

The online version contains supplementary material available at 10.1186/s12917-025-04591-5.

## Background

Diagnostic imaging is evolving rapidly, with significant advancements in artificial intelligence (AI) applications. AI developments in medical imaging include automatic landmark identification in images [1, 2], AI–assisted image analysis [3, 4], automated disease detection [5, 6], and support for clinical decision–making [7]. While significant progress in image processing is particularly evident in human medicine, both human and veterinary medicine utilize the same X–ray imaging–based modalities, such as conventional radiography [8, 9], cone beam computed tomography (CBCT) [4, 10], and fan beam multi–detector computed tomography (MDCT) [2, 11]. Given the advancements in AI–assisted management in human medical imaging – leading to improved accuracy, efficiency, and reliability in medical image analysis [12], as well as better treatment outcomes and more efficient medical practices [13] – similar progress can be anticipated in the field of veterinary medicine.

In veterinary medicine, various diagnostic imaging methods, including ultrasound, conventional radiography, computed tomography (CT), magnetic resonance imaging (MRI), and positron emission tomography (PET), are employed. While PET/CT has been introduced for advanced lameness evaluation in horses [14–16], its use in veterinary practice is limited due to high cost, restricted equipment availability in only a few academic institutions, strict legal regulations, and the need for specialized operator training [10]. MRI is more widely utilized in veterinary medicine, particularly for imaging the head [17] and spine [18] in dogs. In equine practice, high–field MRI has limitations due to its relatively small gantry diameter and the requirement for general anesthesia [19, 20]. However, it has been used for detailed imaging of paranasal sinus cysts [21] and nasal cavity tumors [22]. Low–field MRI, commonly used in equine practice, allows for imaging of standing sedated horses, particularly the distal limb [23]. However, the quality of low–field MRI can be questionable [24] as it may fail to detect small soft tissue lesions [25, 26]. With the advent of dual–energy MDCT, which enables differentiation of soft tissue lesions and bone marrow edema [27, 28], its application in equine imaging [29, 30] has become competitive with low–field MRI. Until recently, MDCT imaging in horses was restricted by gantry size, limiting scans to the peripheral limbs, head, and part of the neck, and requiring general anesthesia. However, recent advancements in equipment and facility modifications have led to the development of equine–specific MDCT systems, allowing imaging under standing sedation [30]. These systems facilitate the imaging of an extended range of limbs and neck structures in standing sedated horses [28, 30, 31] and, under general anesthesia, now permit imaging of previously inaccessible areas such as the entire neck [32] and thoracolumbar spine [33, 34]. Despite substantial agreement between CBCT and MDCT, MDCT is preferred due to the lower image quality and reduced soft tissue contrast of CBCT [35]. Veterinary CT imaging is advancing rapidly, aligning with human medical practices, and MDCT is now routinely used in small animals, exotic pets, and horses for basic imaging, real–time angiography, or 3D rendering techniques [36].

One may observe that in recent decades, CT, MRI, and, to a lesser extent, PET have gained popularity in veterinary clinical practice. However, ultrasound and conventional radiography remain the most widely used modalities, primarily due to concerns regarding general anesthesia risks, equipment availability, and economic factors [10, 20]. As a result, conventional radiography is often the first–line diagnostic imaging modality for the skeleton in both large and small animals, as well as for the thorax and abdomen in small animals. While it is considered complementary to ultrasound for evaluating visceral structures, conventional radiography is frequently recommended as the primary imaging method in small animal and equine practice for a majority of diagnostic applications. Ongoing advancements in conventional radiography, particularly with the integration of bone mineral density (BMD) evaluation, are expected to further enhance and streamline the daily practices of veterinarians working with both small animals [37, 38] and horses [39, 40].

The relevance of BMD assessment in horses stems from its role as a crucial health indicator in the training of racehorses [41, 42] and sport horses [43]. BMD is a key determinant of bone strength, aiding trainers in preventing bone strain and injuries [41–43], while also enabling clinicians to assess fracture risk [44, 45] and monitor the progression of diseases affecting joints, bones, and teeth [39, 40]. As a result, several noninvasive methods for BMD evaluation have been investigated. Some attempts have been made to assess BMD using radiation–free modalities such as quantitative ultrasound [46] and single–photon absorptiometry [47]. Quantitative ultrasonography measures the speed and attenuation of sound waves in bone [46], while the single–photon absorption method determined BMD by measuring the amount of gamma rays emitted by isotopes that pass through the bone [47]. However, BMD has been extensively investigated using X–ray beam attenuation measurements including single–energy X–ray computed digital absorptiometry (CDA) [37–41, 48–51] or dual–energy X–ray absorptiometry (DXA) [41, 47, 49, 51]. DXA determines BMD by passing two X–ray beams with different energy levels through the patient’s body and measuring the differences in absorption by the bone [41, 47, 49, 51]. In contrast, CDA uses a single X–ray beam that passes through both the patient’s body and a density standard of known properties, determining BMD based on the similarities in beam absorption between the bone and the density standard [39, 41, 48–50]. While DXA is the preferred method for BMD evaluation in humans [52–54], its clinical application in horses is significantly limited due to the absence of equine–specific systems, the limited availability of human systems adopted for equine use, and the requirement for general anesthesia during assessment [41, 50]. On the other hand, the clinical application of CAD is restricted by the lack of the commercially available density standards, reference X–ray tube settings, and dedicated software that would facilitate its routine use in veterinary practice. Consequently, considerable efforts have been directed towards advancing the clinical applications of MDCT [32, 41, 51, 55], particularly under standing sedation, as well as enhancing conventional radiography supported by user–friendly CAD [39, 41, 48, 50, 56, 57] for BMD assessment.

In MDCT–derived images, the linear attenuation coefficient is converted to the Hounsfield scale [49, 58], where radiodensity is defined as -1000 Hounsfield unit (HU) for air, 0 HU for distilled water, 20–100 HU for soft tissue, up to 1000 HU for bone, and 2000 HU for dense bone or tooth [59]. Voxel HU values can be transformed into BMD values (r = a × HU + b) [54], which determine the attenuation properties of bones expressed in volumetric BMD (vBMD) [37, 45]. In CDA method, the mass attenuation coefficient [60] of a density standard is used to compare the pixel brightness (PB) of the density standard and bone on a radiograph [50]. By comparing the PB of bone to that of the density standard, the relative BMD (rBMD) [37, 38] can be calculated and expressed as the brightness/darkness index (BDI) [50], the number of pixels (NP) [39, 61] or percentage of pixels [37, 40] within a specific PB range, or the HU equivalent calculated based on the mean standard attenuation [38]. Regardless of the form in which it is expressed, these measures enable the calibration of grayscale values on radiographs, correlating bone attenuation properties to the density standard. This allows for the comparison of bone characteristics across different radiographs [39–41, 48, 50, 56, 57, 61]. Unlike DXA, which is non–portable and impractical for use with standing, conscious horses [41], the CDA method requires only conventional radiography, an appropriate density standard, and software for processing the radiograph. Therefore, it can be easily applied, even in field equine practice [39–41, 50]. As the X–ray beam attenuation obtained from radiographs also depends on the intensity of the radiographic exposure (kV) and the quantity of the beam (mAs) [50], aligning the attenuation properties of the density standard with the exposure settings will expand the clinical applicability of the CDA method. To achieve this alignment, the k–means clustering algorithm, an unsupervised machine learning (ML) technique, was employed. K–means clustering partitions *n* observations into *k* clusters based on proximity to the nearest mean [62] and has already been applied to equine datasets to cluster MDCT volumetric data, leading to the identification of age–related changes in paranasal sinuses [63] and incisor teeth [2]. K–means clustering is one of the most well–known and widely used methods of vector quantization, which has been extensively applied to human medical imaging datasets, including PET/CT [64, 65], MRI [66, 67], and MDCT, particularly for clustering bones with low and high BMD [68, 69].

Hence, the primary aim of this study was to investigate whether the k–means clustering algorithm, using CDA data, can differentiate cortical and trabecular bone in healthy horses. Subsequently, the study aims to identify the optimal CDA protocol for effective bone type clustering, including the selection of density standards and X–ray tube settings. Finally, the study aims to determine whether the k–means clustering algorithm, using CDA data from the selected protocol, can effectively differentiate cortical and trabecular bone in horses affected by osteoarthritis.

## Methods

### Study design

The three–stage study was designed to optimize the CDA method based on rBMD of two bone types, clustered using the k–means clustering algorithm.

**Fig. 1:**
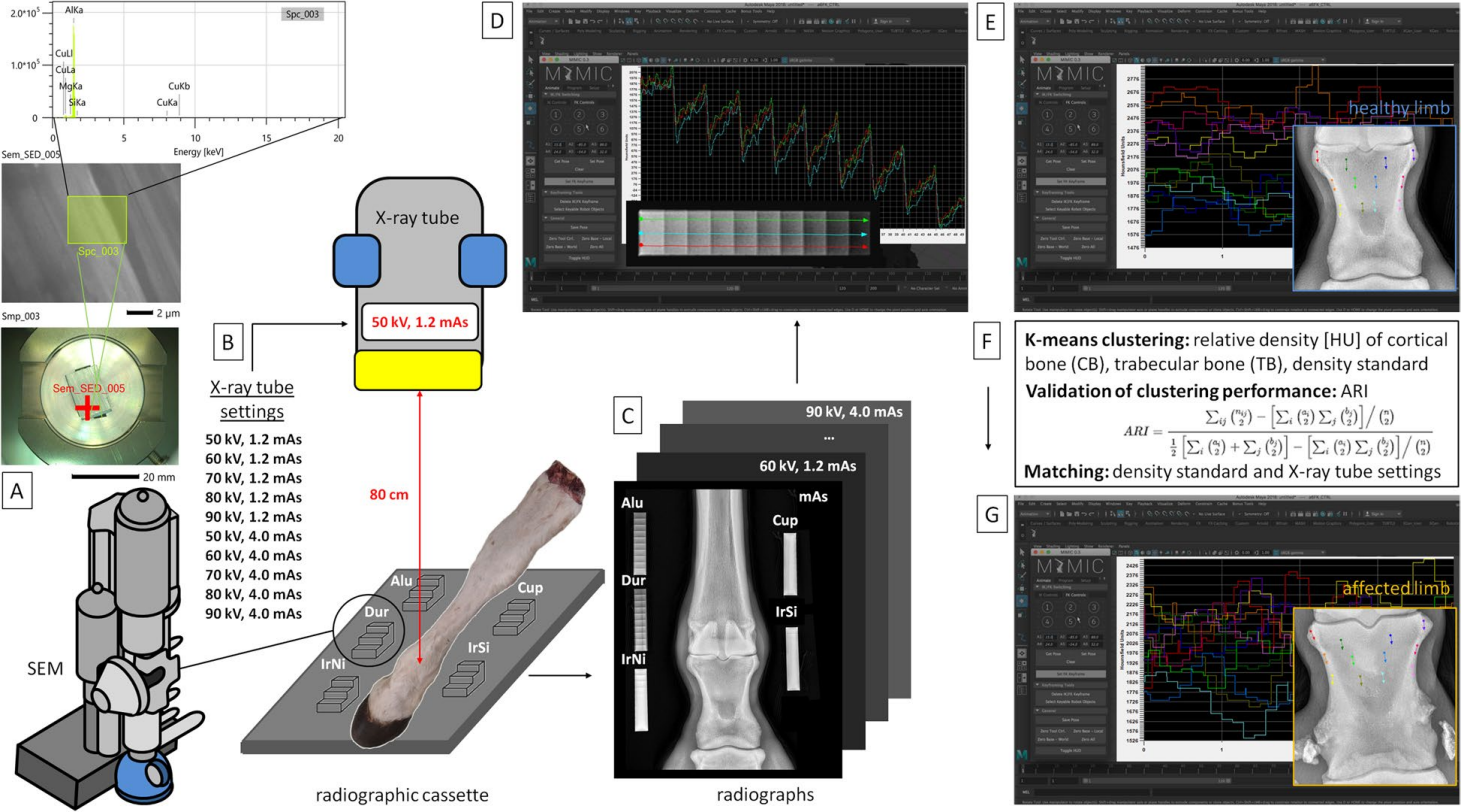
Study design including three stages. First stage: Five metals–made density standards (Alu, pure aluminum; Dur, aluminum alloy (duralumin); Cup, cuprum alloy; IrNi, iron–nickel alloy; IrSi, and iron–silicon alloy) evaluation under scanning electron microscope (SEM) (**A**); Second stage: Radiographs of healthy limbs – acquisition and processing with five density standards under ten X–ray tube settings; dataset were then clustered to select the optimal computed digital absorptiometry (CDA) protocol (**B**); Third stage: Radiographs of affected limbs – acquisition and processing with one density standard under selected X–ray tube setting; dataset were then clustered to test the optimal CDA protocol (**C**)

In the first stage, the five density standards were characterized in detail using the basic measurements and elements composition (Fig. 1A).

In the second stage, radiographs of the long pastern bone (the proximal phalanx) from six equine healthy cadaver front limbs and five density standards were taken under ten different X–ray tube settings. The limbs were collected postmortem at a commercial slaughterhouse. Radiographs were obtained in a shielded radiographic room with lead walls, lead doors, and lead glass, which separated the radiographic room from the control room. To ensure radiation safety, radiographs were taken in the closed radiographic room with no personnel present, and the X–ray exposure was activated remotely from the shielded control room. Each radiograph included five density standards and one cadaver limb. The rBMD was extracted from the cortical bone (CB) and trabecular bone (TB) of each healthy long pastern bone, and the k–means clustering algorithm was used to differentiate CB and TB. A total of 50 CDA parameters (five density standards $\times$ ten X–ray tube settings) were investigated (Fig. 1B).

In the third stage, radiographs of the long pastern bone from six equine affected cadaver front limbs and one selected density standard (described below) were taken under the optimal X–ray tube setting. Each radiograph contained one density standard and one cadaver limb. The rBMD was extracted for both CB and TB, and k–means clustering algorithm was used to differentiate CB and TB (Fig. 1C).

### Density standards characteristics

**Fig. 2:**
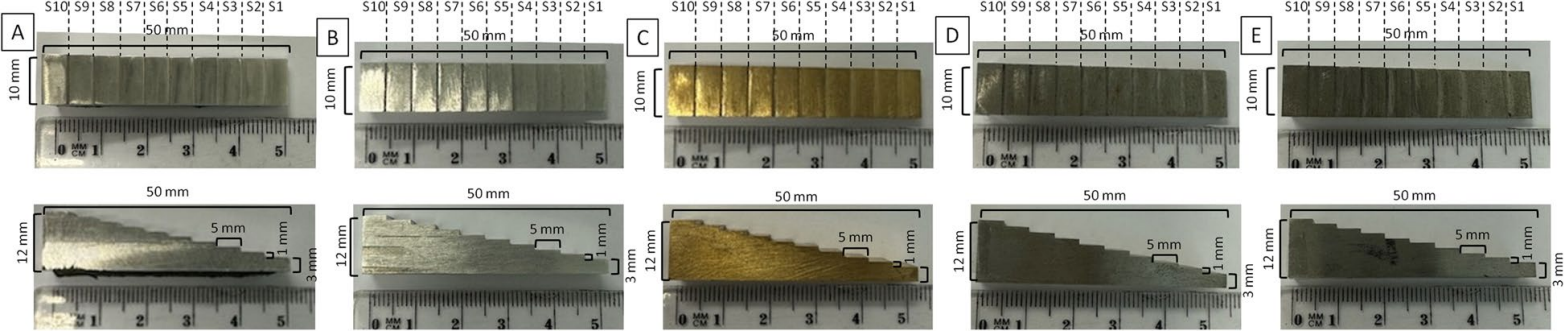
Two projections of density standards made of pure aluminum (Alu) (**A**), aluminum alloy (duralumin, Dur) (**B**), cuprum alloy (Cup) (**C**), iron–nickel alloy (IrNi) (**D**), and iron–silicon alloy (IrSi) (**E**) – the perpendicular to the base projection and lateral projection. Both projections show the dimensions of each density standard and have marked ten steps (S1–S10)

The subject of the study consisted of five density standards made from different metals: pure aluminum (Alu; Fig. 2A), aluminum alloy (duralumin, Dur; Fig. 2B), cuprum alloy (Cup; Fig. 2C), iron–nickel alloy (IrNi; Fig. 2D), and iron–silicon alloy (IrSi; Fig. 2E). The density standards were shaped as irregular cuboid with 10 steps (S1–S10), each decreasing the height of the cuboid.

The dimensions of each density standard were as follows: 50 mm length of the base, 10 mm width, 12 mm height at the highest point, and 3 mm height at the lowest point. Each step measured 10 mm in width, 5 mm in length, and 1 mm in height, except for the lowest step, which had a height of 3 mm. Therefore, the volume of each density standard was 3750 mm^3^. The mass and density of each density standard are summarized in Table 1.

**Table 1:** The mass and density of each density made of pure aluminum (Alu), aluminum alloy (duralumin, Dur), cuprum alloy (Cup), iron–nickel alloy (IrNi), and iron–silicon alloy (IrSi)

Density standard	Alu	Dur	Cup	IrNi	IrSi
Mass [g]	10.13	10.46	33.45	29.63	28.88
Density [g/cm^3^]	2.70	2.79	8.92	7.90	7.70

The scanning electron microscope (SEM) (JCM–7000 NeoScope™ Benchtop SEM, JEOL, Tokyo, Japan) was used to determine the elements composition of each density standard. The macroscopic samples (Smp) were positioned under the SEM lens, while microscopic samples (Sem_SED) were imaged using acceleration voltage of 15.00 kV, a process time of T2, and a live time of 30.00 s. The intensity for each energy $\in <0;20>$ [keV] was recorded and displayed on the intensity versus energy chart. Based on the intensities of characteristic energies, the mass percentage (Mass%) and atomic percentage (Atom%) of each element were calculated.

### Radiograph acquisition

The radiographs were acquired using an X–ray tube (Orange 9020HF, Ecoray Co., Seoul, Korea), a radiographic cassette (Saturn 8000, Vievorks Co., Seoul, Korea), and a portable computer (HP Inc UK Ltd, Reading, UK). The distance between the X–ray tube and the radiographic cassette was always 80 cm. The radiographs were acquired using the DxWorks software (Vievorks Co., Ltd., Seoul, Korea) as DISOM files. Each equine cadaver front limb was positioned in the middle of a radiographic cassette, with the center of the X–ray beam focused on the middle of the long pastern bone. Each long pastern bone was imaged in the dorso–palmar projection. In the second stage of the study, five density standards were positioned next to each other and next to the cadaver limb, perpendicular to the surface of the radiographic cassette. In the third stage of the study, one density standard was similarly positioned next to the cadaver limb. Each density standard was imaged in the top–bottom projection, with the long base placed on the cassette and the steps of the density standard facing the X–ray tube. In the second stage of the study, ten radiographs were acquired for each limb and set of density standards, resulting in 50 radiographs. In the third stage of the study, one radiograph was acquired for each limb and selected density standards, resulting in radiographs. In total, 60 radiographs were analyzed. For the third stage of the study, the X–ray tube setting was selected from the results, while the X–ray tube settings used in the second stage of the study are summarized in Table 2.

**Table 2:** The X–ray tube settings, included the current and exposure time (mAs) and voltage (kV), used for the imaging of five density standards made of different metals

**mA /kV**	50	60	70	80	90
1.2	50, 1.2	60, 1.2	70, 1.2	80, 1.2	90, 1.2
4.0	50, 4.0	60, 4.0	70, 4.0	80, 4.0	90, 4.0

### Radiograph processing

The Materialise interactive medical image control system (MIMICS) software (Materialise HQ, Leuven, Belgium) was used to analyze the attenuation of the X–ray beam passing through each long pastern bone and each density standard.

For each dorso–palmar projection of the long pastern bones, the attenuation of the X–ray beam was measured using six lines positioned parallel to the long axis of the bone on CB and six measuring lines positioned parallel to the long axis of the bone on TB. Bone type was identified based on radiodensity and anatomical location. The CB was identified marginally as an area of higher radiodensity (opacity) relative to the TB, which was identified centrally as an area of less radiodensity (lucency). The radiographs of equine limbs were assessed and graded for signs of osteoarthritis by an experienced equine surgeon, a Polish board–certified equine disease specialist (PCED) (B.T.), and a third-year resident specializing in Polish board–certified veterinary diagnostic imaging (PCVDI) (M.D.), independently. Any disagreements were resolved by a Polish board–certified veterinary diagnostic imaging (PCVDI) radiologist (T.J.). Only the radiographic signs of osteoarthritis were considered in this study. The underlying causes of the visible radiological signs were not addressed.

**Fig. 3:**
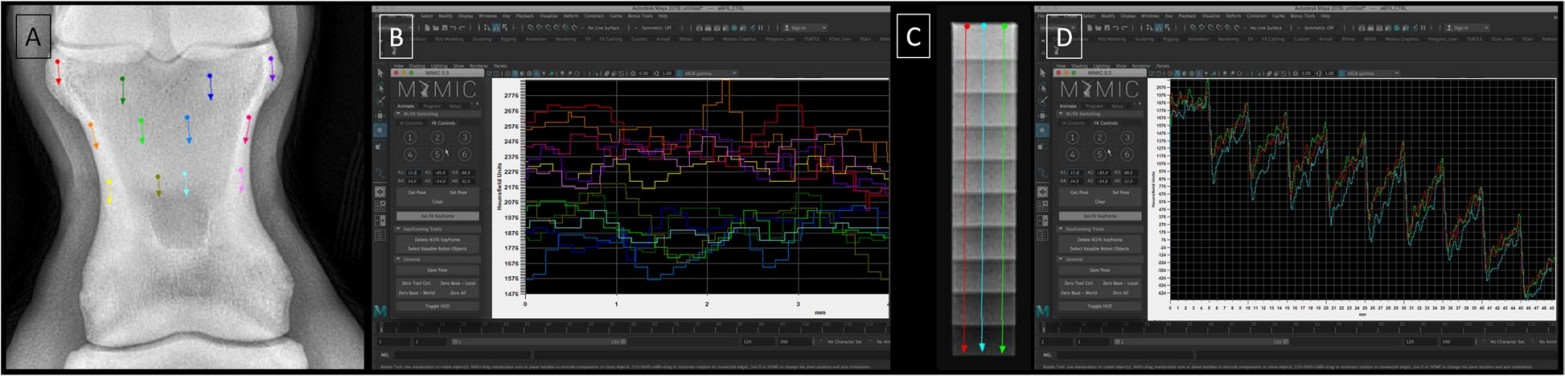
Measuring lines (**A**,**C**) and related charts of relative density [HU] versus distance [mm] (**B**,**D**) displayed for evaluation of bone (**A**,**B**) and density standard (**C**,**D**). The colors of the measurement lines correspond to the colors on the charts

For each bone, twelve measuring lines were marked in different colors. The measuring lines were positioned at the proximal part of the long pastern bone near the articular surface, at the proximal one–third of the long pastern bone, and at the mid–height of the long pastern bone, on the right and left sides (Fig. 3A). The rBMD [HU] for subsequent millimeters of each bone type was calculated and displayed on the relative density [HU] versus distance [mm] chart (Fig. 3B). Numerical rBMD values was extracted for each measuring line.

For a top–bottom projection of the density standards, the attenuation of the X–ray beam was measured using three parallel measuring lines, which also were marked by different colors. These measuring lines represented the lateral, middle, and medial aspect of each density standard (Fig. 3C). The relative density [HU] for subsequent millimeters of each standard density was calculated and displayed on the relative density [HU] versus distance [mm] chart. Based on the three measuring lines of attenuation, three HU values were calculated for each step of density standard (Fig. 3D).

### K–means clustering analysis of relative density data

The input data sets for the k–means clustering analysis were the rBMD of two bone types, CB and TB, as well as the relative densities of the density standards. The k–means clustering analysis was performed in Python using the machine learning package sci–kit–learn [70]. The script for running the program using the k–means method included the following parameters: n_clusters = 2, init = "k–means + + ", n_init = 50, max_iter = 500, and random_state = 42. The n_clusters parameter specifies the number of clusters to form and the number of centroids to generate. The init parameter defines the initialization method, where the initial cluster centroids are selected based on an empirical probability distribution of the points’ contribution to the overall inertia. The n_init parameter indicates how many times the k–means algorithm is run with different centroid seeds. The max_iter parameter sets the maximum number of iterations for a single run of the k–means algorithm. Finally, the random_state parameter controls the random number generation for centroid initialization [71].

The first step involved normalizing the data to ensure feature values were on the same scale. Normalization was performed using the MinMaxScaler class [71] from the sci–kit–learn package. The dimensionality of the bone data (CB and TB) was reduced using principal component analysis [72]. No dimensionality reduction was applied to the density standards data.

In the second step, the k–means class was used to perform clustering [73]. The basic k–means clustering algorithm was implemented to select k centroids, where k was equal to the number of specified clusters. To distinguish between bones, 2 clusters were set, while 10 clusters were set to determine the best CDA parameters. Centroids were data points representing the center of a cluster. Each data point was assigned to its nearest centroid. The average of all points within each cluster was then calculated to determine a new centroid. Silhouette coefficients (SC) [74] were used to evaluate the selection of the appropriate number of clusters. Silhouette coefficient values range from -1 to 1, with larger values indicating that samples are closer to their own clusters than to other clusters.

In the third step, clustering validity was assessed using the adjusted rand index (ARI) metric. The ARI uses true cluster assignments to measure the similarity between true and predicted labels. The ARI output values range from -1 to 1, with a score close to 0.0 indicating random assignments and a score close to 1 indicating perfectly labeled clusters.

### Statistical analysis

The relative density values measured for each of S1–S10 of each density standard were tested for data distribution using a Shapiro–Wilk normality test. Since the data did not follow a normal distribution, the relative density of subsequent density standards was compared using the Kruskal–Wallis test, followed by the post–hoc Dunn’s multiple comparisons test. The significance level was set at *p* < 0.05. Data are presented in plots showing the median and range, with the *p* value displayed on each plot. The relative density of subsequent density standards was compared only for the highest step (S10).

The similarity between the relative density values under different X–ray tube settings was tested using linear regression for Alu, Dur, Cup, IrNi, and IrSi density standards separately. The regression line was determined based on all relative density values for the S1–S10. The regression equations and r^2^ values were calculated for the 45 data pairs resulting from combinations of mAs (1.2; 4.0) and kV (50; 60; 70; 80; 90). The significance level was set at *p* < 0.05. When the differences between the slopes were not significant (*p* > 0.05), a single slope for both datasets was calculated, and the intercepts were compared. When the differences between the intercepts were not significant (*p* > 0.05), a single intercept for both datasets was calculated. The significance levels for each similarity measure were summarized in appropriate tables.

The rBMD measured measured for each long pastern bone were tested for data distribution using a Shapiro–Wilk normality test. Since the data did not follow a normal distribution, the rBMD values for CB and TB was compared using the Mann–Whitney test. The significance level was set at *p* < 0.05. Data are presented in plots showing the median and range, with the *p* value displayed on each plot.

## Results

### The material characteristic of density standards

**Fig. 4:**
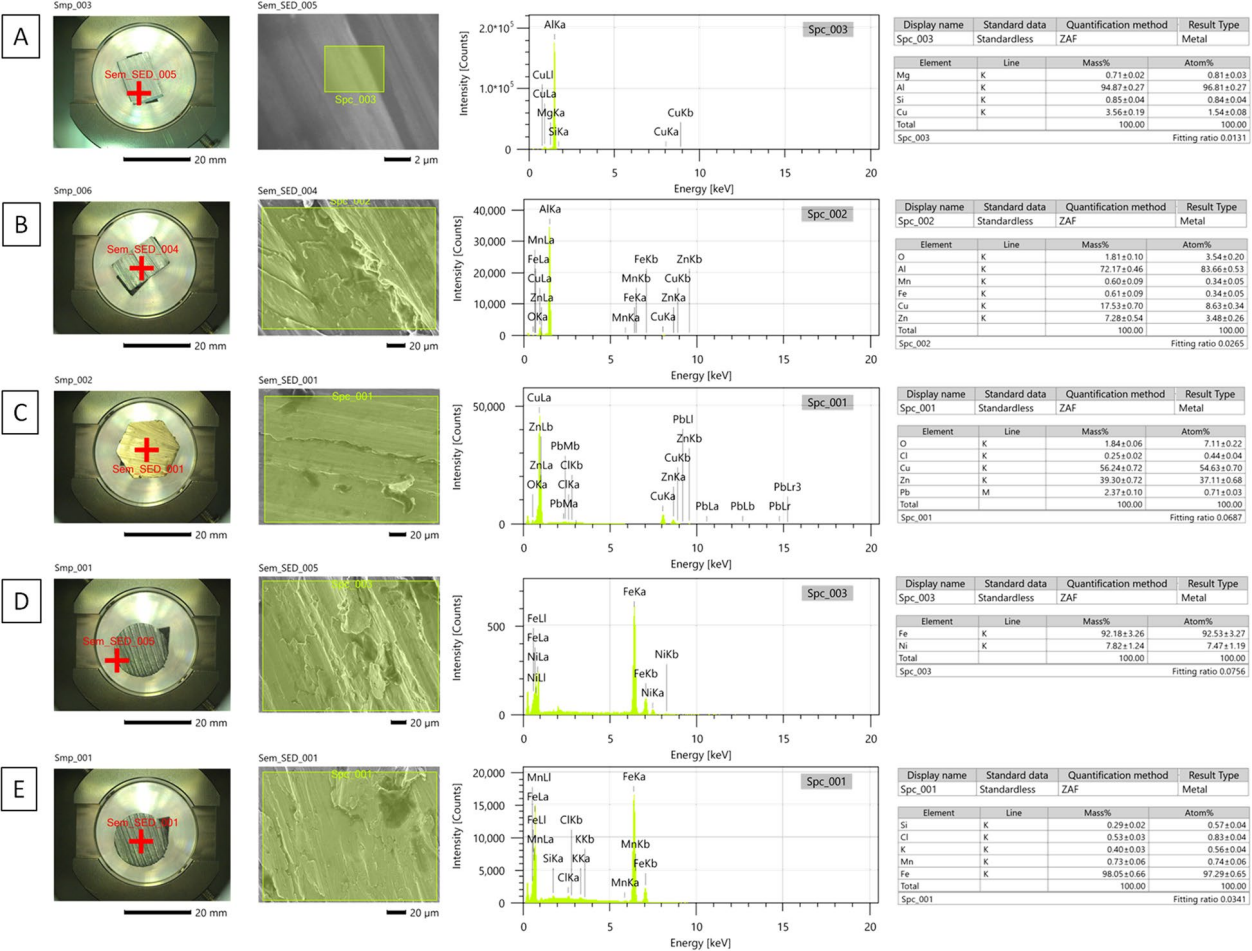
Composition of density standards made of pure aluminum (Alu) (**A**), aluminum alloy (duralumin, Dur) (**B**), cuprum alloy (Cup) (**C**), iron–nickel alloy (IrNi) (**D**), and iron–silicon alloy (IrSi) (**E**) evaluated under a scanning electron microscope (SEM). Subfigures show the macroscopic sample (Smp), microscopic sample (Sem_SED), intensity versus energy chart, as well as mass percentage (Mass%) and atomic percentage (Atom%) corresponding to elements composition

For the Alu density standard, the highest intensity corresponded to aluminum (Al), lower intensity to copper (Cu) and silicon (Si), and the lowest intensity to magnesium (Mg) (Fig. 4A). For the Dur density standard, the highest intensity corresponded to Al, lower intensity to Cu, zinc (Zn), oxygen (O), and iron (Fe), and the lowest intensity to manganese (Mn) (Fig. 4B). For the Cup density standard, the highest intensity corresponded to Cu, lower intensity to Zn, lead (Pb), and O, and the lowest intensity to chlorine (Cl) (Fig. 4C). For the IrNi density standard, higher intensity corresponded to Fe and lower intensity to nickel (Ni) (Fig. 4D). For the IrSi density standard, higher intensity corresponded to Fe and lower intensity to Si (Fig. 4E). The Mass% and Atom% of elements in each density standard are summarized in Table 3.

**Table 3:** A mass percentage (Mass%) and atomic percentage (Atom%) of elements of each density standard made of the pure aluminum (Alu), aluminum alloy (duralumin, Dur), cuprum alloy (Cup), iron–nickel alloy (IrNi), and iron–silicon alloy (IrSi)

Density standard	Alu	Dur	Cup	IrNi	IrSi
Mass%	Al 94.87 ± 0.27 Cu 3.56 ± 0.19 Si 0.85 ± 0.04 Mg 0.71 ± 0.02	Al 72.17 ± 0.46 Cu 17.53 ± 0.70 Zn 7.28 ± 0.54 O 1.81 ± 0.10 Fe 0.61 ± 0.09 Mn 0.60 ± 0.09	Cu 56.24 ± 0.72 Zn 39.30 ± 0.72 Pb 2.37 ± 0.10 O 1.84 ± 0.06 Cl 0.25 ± 0.02	Fe 92.18 ± 3.26 Ni 7.82 ± 1.24	Fe 97.93 ± 0.65 Si 2.07 ± 0.05
Atom%	Al 96.81 ± 0.27 Cu 1.54 ± 0.08 Si 0.85 ± 0.04 Mg 0.81 ± 0.03	Al 83.66 ± 0.53 Cu 8.63 ± 0.34 Zn 3.48 ± 0.26 O 3.54 ± 0.20 Fe 0.34 ± 0.05 Mn 0.34 ± 0.05	Cu 54.63 ± 0.70 Zn 37.11 ± 0.68 Pb 0.71 ± 0.03 O 7.11 ± 0.22 Cl 0.44 ± 0.04	Fe 92.53 ± 3.27 Ni 7.47 ± 1.19	Fe 95.98 ± 0.63 Si 4.02 ± 0.10

*Al* aluminum, *Cu* copper, *Si* silicon, *Mg* magnesium, *Zn* zinc, *O* oxygen, *Fe* iron, *Mn* manganese, *Pb* lead, *Cl* chlorine, *Ni* nickel

### The X–ray beam attenuation characteristics of density standards

**Fig. 5:**
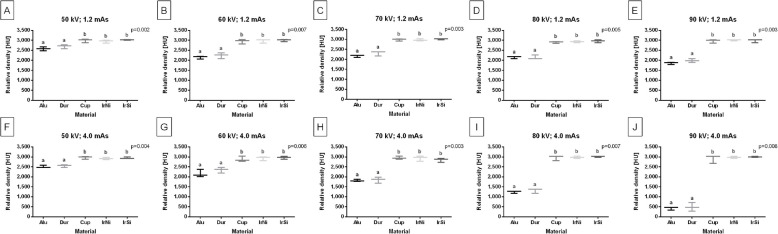
Composition of the relative density [HU] of the highest step (S10) of density standard between Alu, Dur, Cup, IrNi, and IrSi density standards. Comparison made for all studied X–ray tube settings (**A**–**J**), independently. Data are represented by the median and range. Lowercase letters indicate differences between materials for *p* < 0.05

The relative density of the highest step (S10) of density standard differed between materials regardless of the X–ray tube settings. Across all studied X–ray tube settings, the relative density was higher for the Cup, IrNi, and IrSi density standards compared to for the Alu and Dur density standards (*p* ≤ 0.002). However, the post–hoc test shown no differences in relative density among the Cup, IrNi, and IrSi density standards, nor between the Alu and Dur density standards (Fig. 5A–J).

For the Alu density standard, X–ray beam attenuation and relative density decreased step by step across all X-ray tube settings (Additional File 1 A–Y). However, under certain settings, specific steps were overexposed (Table 4). The X–ray beam attenuation slopes remained consistent for most settings, except for 70 kV, 4.0 mAs; 80 kV, 4.0 mAs; and 90 kV, 4.0 mAs, which showed differences. The intercepts of the X–ray beam attenuation line differed across settings, except between 60 kV, 1.2 mAs and 70 kV, 1.2 mAs, where no differences were observed (Additional File 2).

**Table 4:** The specifications of the density standards, X-ray settings, and specific steps (S1-S10) where overexposure was observed

Density standards	X–ray settings	Steps	Exposition issue	Supporting figures
Alu	80 kV, 4.0 mAs	S1-S3	overexposed	Additional File 1X
	90 kV, 4.0 mAs	S1-S7	overexposed	Additional File 1Y
Dur	80 kV, 4.0 mAs	S1-S3	overexposed	Additional File 3X
	90 kV, 4.0 mAs	S1-S7	overexposed	Additional File 3Y

*Alu* pure aluminum, *Dur* duralumin

For the Dur density standard, X–ray beam attenuation and relative density decreased step by step across all X-ray tube settings (Additional File 3 A–Y). As with the Alu density standard, certain settings led to overexposure of specific steps (Table 4). The slopes of the X-ray beam attenuation line remained consistent across most settings, except for 60 kV, 4.0 mAs; 70 kV, 4.0 mAs; 80 kV, 4.0 mAs; and 90 kV, 4.0 mAs, which showed differences. The intercepts of the X–ray beam attenuation line varied across settings, except for three pairs (60 kV, 1.2 mAs vs. 70 kV, 1.2 mAs; 60 kV, 1.2 mAs vs. 60 kV, 4.0 mAs; 70 kV, 1.2 mAs vs. 60 kV, 4.0 mAs), where no differences were observed (Additional File 4).

For the Cup density standard, X–ray beam attenuation and relative density did not consistently decrease across consecutive steps for all X-ray tube settings (Additional File 5A–Y). Both the slopes and intercepts of the X–ray beam attenuation line showed no differences between the studied X–ray tube settings (Additional File 6).

For the IrNi density standard, the X–ray beam attenuation and relative density did not consistently decrease across consecutive steps for certain settings (50 kV, 1.2 mAs / 50 kV, 4.0 mAs / 60 kV, 1.2 mAs / 60 kV, 4.0 mAs / 70 kV, 1.2 mAs / 70 kV, 4.0 mAs) (Additional File 7 A–C, F–H, P–R, U–W). However, a partial decrease was observed for other settings (80 kV, 1.2 mAs / 80 kV, 4.0 mAs / 90 kV, 1.2 mAs / 90 kV, 4.0 mAs) (Additional File 7 D,E,I,J,S,T,X,Y). The slopes and intercepts of the X–ray beam attenuation line remained consistent across all studied X-ray tube settings, except 90 kV, 1.2 mAs; 80 kV, 4.0 mAs; and 90 kV, 4.0 mAs (Additional File 8).

For the IrSi density standard, the X–ray beam attenuation and relative density did not consistently decrease across consecutive steps for certain settings (50 kV, 1.2 mAs / 50 kV, 4.0 mAs / 60 kV, 1.2 mAs / 60 kV, 4.0 mAs / 70 kV, 1.2 mAs / 70 kV, 4.0 mAs) (Additional File 9 A–C, F–H, P–R, U–W). However, a partial decrease was observed for other settings (80 kV, 1.2 mAs / 80 kV, 4.0 mAs / 90 kV, 1.2 mAs / 90 kV, 4.0 mAs) (Additional File 9 D,E,I,J,S,T,X,Y). The slopes of the X–ray beam attenuation line were similar for most low–energy setting pairs (50 kV, 60 kV, 70 kV) except 90 kV, 1.2 mAs; 80 kV, 4.0 mAs; and 90 kV, 4.0 mAs. The intercepts of the X–ray beam attenuation line were consistent between certain pairs (50 kV, 1.2 mAs and 80 kV, 1.2 mAs; 50 kV, 1.2 mAs and 60 kV, 4.0 mAs; 50 kV, 1.2 mAs and 70 kV, 4.0 mAs; as well as between 70 kV, 1.2 mAs and 90 kV, 1.2 mAs) (Additional File 10).

### The X–ray beam attenuation characteristics of cortical and trabecular bone of healthy limbs

**Fig. 6:**
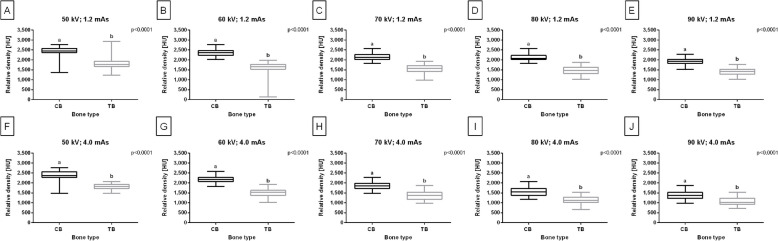
Composition of the relative bone mineral density (rBMD) [HU] of the cortical bone (CB) and trabecular bone (TB) of healthy horses limbs. Comparison made for all studied X–ray tube settings (**A**–**J**), independently. Data are represented by the median and range. Lowercase letters indicate differences between materials for *p* < 0.05

In healthy horses, the rBMD of the CB and TB differed between bone types, regardless of the X–ray tube settings (*p* < 0.0001). For all studied X–ray tube settings, the rBMD was higher (*p* < 0.0001) for CT than for TB (Fig. 6A–J).

**Fig. 7:**
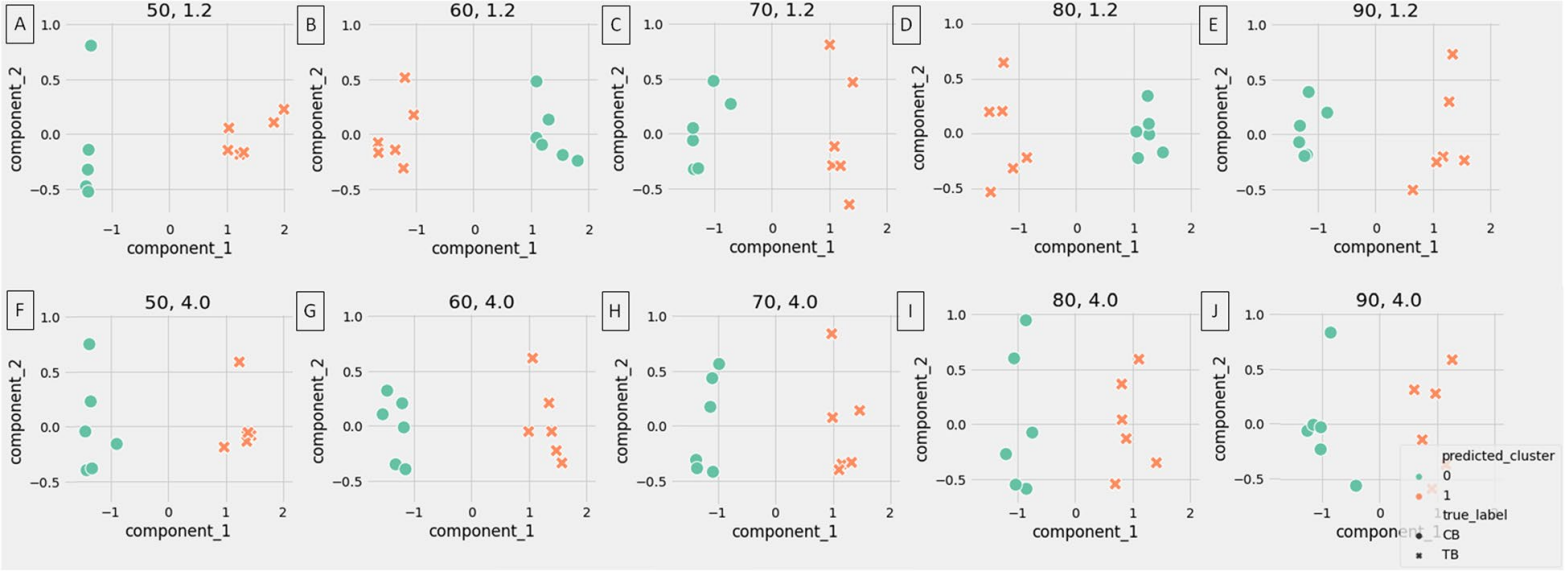
Clustering of the cortical bone (CB) and trabecular bone (TB) of healthy horses limbs. Clustering made for all studied X–ray tube settings (**A**–**J**), independently

The rBMD values of CB and TB allowed for the clustering of bone types of healthy limbs into two classes, displayed for all X–ray tube settings (Fig. 7A–J). The metrics for cluster cohesion and separation were arranged hierarchically, with samples being closer to their clusters (SC closer to 1). The highest SC was observed for 60 kV and 4.0 mAs X–ray tube settings. When evaluating clustering using truth labels, the similarity between true and predicted labels was full (ARI = 1) for all X–ray tube settings. Therefore, each exposure setting allowed for the separation of CB and TB in the radiographs (Table 5).

**Table 5:** Clustering of the cortical bone (CB) and trabecular bone (TB) of healthy horses limbs. The silhouette coefficient (SC), adjusted rand index (ARI), Intertia, and coordinates of cluster centers (x1, y1, x2, y2) measured for clustering of bone type for ten X–ray tube settings. Scores were ranked from best fit (SC closer to 1) to least fit (SB closer to -1) into its assigned cluster

Settings	SC	ARI	Intertia	× 1	y1	× 2	y2
60 kV, 4.0 mAs	0.815	1.000	1.426	-1.309	-0.024	1.309	0.024
60 kV, 1.2 mAs	0.813	1.000	1.540	1.348	0.005	-1.348	-0.005
50 kV, 4.0 mAs	0.810	1.000	1.717	-1.296	-0.005	1.296	0.005
80 kV, 1.2 mAs	0.801	1.000	1.578	1.242	0.006	-1.242	-0.006
50 kV, 1.2 mAs	0.780	1.000	2.926	-1.399	0.021	1.399	-0.021
90 kV, 1.2 mAs	0.772	1.000	1.925	-1.176	0.032	1.176	-0.032
70 kV, 4.0 mAs	0.748	1.000	2.396	-1.171	0.007	1.171	-0.007
70 kV, 1.2 mAs	0.745	1.000	2.485	-1.183	0.014	1.183	-0.014
90 kV, 4.0 mAs	0.653	1.000	2.841	-0.946	-0.011	0.946	0.011
80 kV, 4.0 mAs	0.636	1.000	3.399	-0.956	0.007	0.956	-0.007

**Fig. 8:**
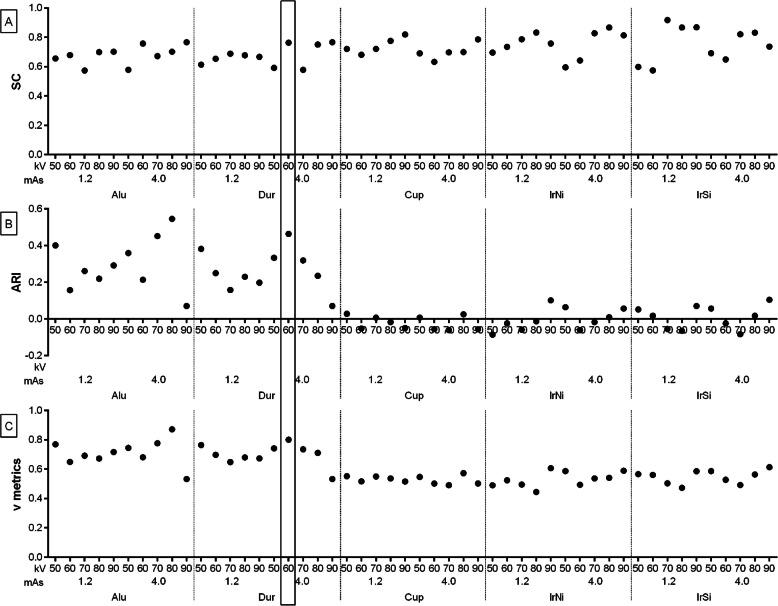
Clustering of the computed digital absorptiometry (CDA) parameters concerning five density standards (Alu, Dur, Cup, IrNi, and IrSi) and ten X–ray tube settings (50 kV, 1.2 mAs; 60 kV, 1.2 mAs; 70 kV, 1.2 mAs; 80 kV, 1.2 mAs; 90 kV, 1.2 mAs; 50 kV, 4.0 mAs; 60 kV, 4.0 mAs; 70 kV, 4.0 mAs; 80 kV, 4.0 mAs; and 90 kV, 4.0 mAs). The silhouette coefficient (SC) (**A**), adjusted rand index (ARI) (**B**), and v metrics (**C**) values are displayed for each matching. Matching with the highest values of all metrics is marked with solid box

The relative density values of the density standards returned clustering metrics ranging from 0.574 to 0.918 for SC (Fig. 8A), from -0.086 to 0.546 for ARI (Fig. 8B), and from 0.445 to 0.872 for v metrics (Fig. 8C). The Dur density standard and 60 kV, 4.0 mAs X–ray tube settings corresponded to the highest values across all metrics. Thus, 60 kV, 4.0 mAs settings in the CDA protocol were consider optimal for healthy distal limb imaging.

### The X–ray beam attenuation characteristics of cortical and trabecular bone of affected limbs

**Fig. 9:**
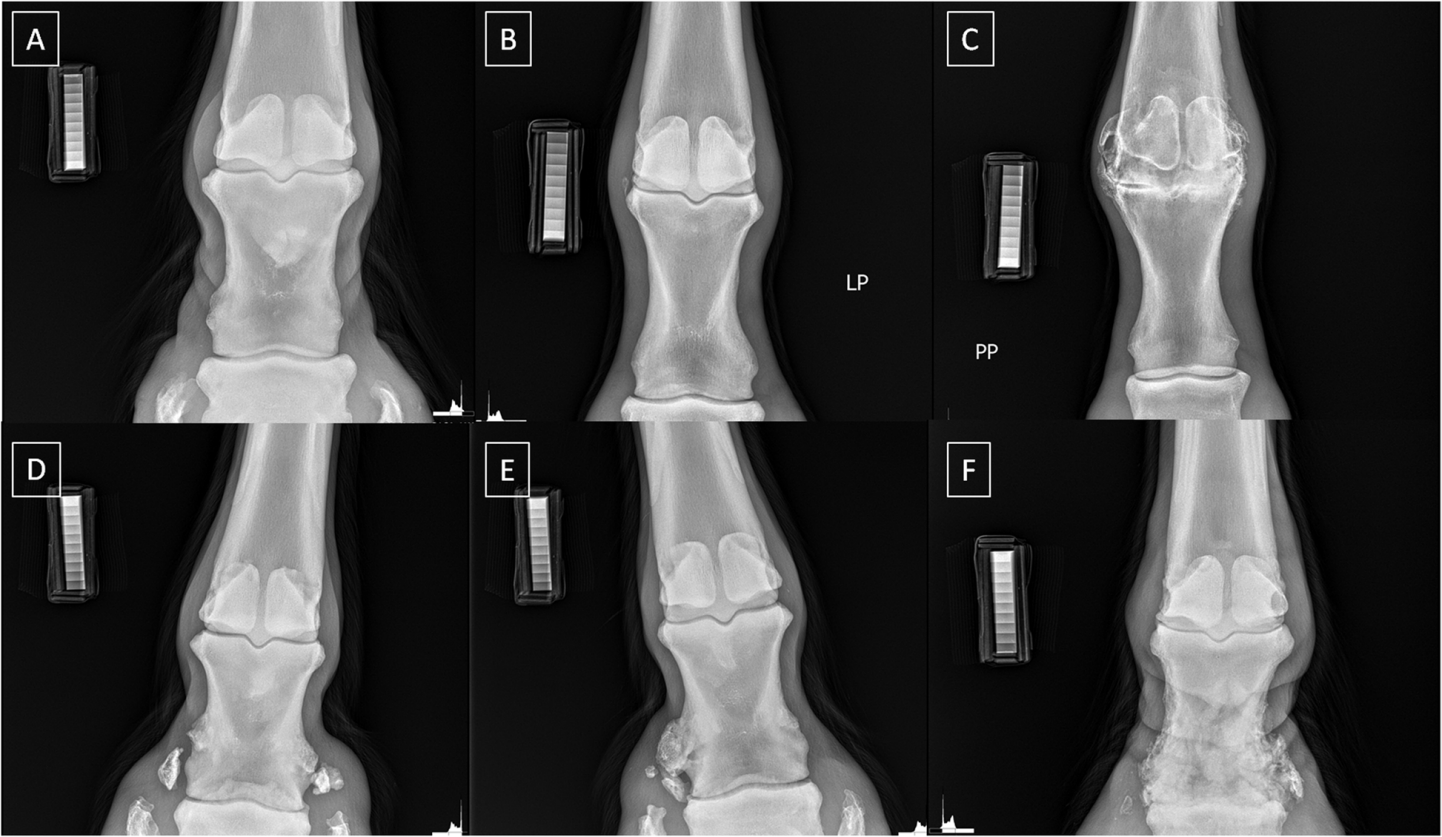
Radiographs of equine limbs classified radiologically as affected with osteoarthritis (OA). Radiographs represented minor OA of fetlock joint (**A**), mild OA of fetlock joint (**B**), severe OA of fetlock joint (**C**), mild OA of pastern joint (**D**), moderate OA of pastern joint (**E**), and severe OA of pastern joint (**F**)

Since the Dur density standard matched with the 60 kV and 4.0 mAs X–ray tube setting was considered optimal, only these CDA parameters were used for affected limbs evaluation. The radiographs of affected limbs represented long pastern bone with the radiographic signs of chronic osteoarthritis (OA) in the proximal aspect (fetlock joint; Fig. 9A–C) and in the distal aspect (pastern joint; Fig. 9D–F). The radiographic signs of degenerative changes in the affected joints included a narrow and irregular joint space, characterized by thin and uneven lucency between the adjacent cortical bones. Additionally, osteophytes and enthesiophytes (bone outgrowths on the surface of the cortical bone) were observed. Subchondral bone sclerosis (an area of increased opacity within the cortical and subcortical bone) and intra-articular mineralization (severely increased opacity inside the joint space) were also noted [75, 76]. The severity of radiographic signs of OA gradually increased from minor (Fig. 9A), mild (Fig. 9B,C), moderate (Fig. 9E), to severe (Fig. 9C,F).

**Fig. 10:**
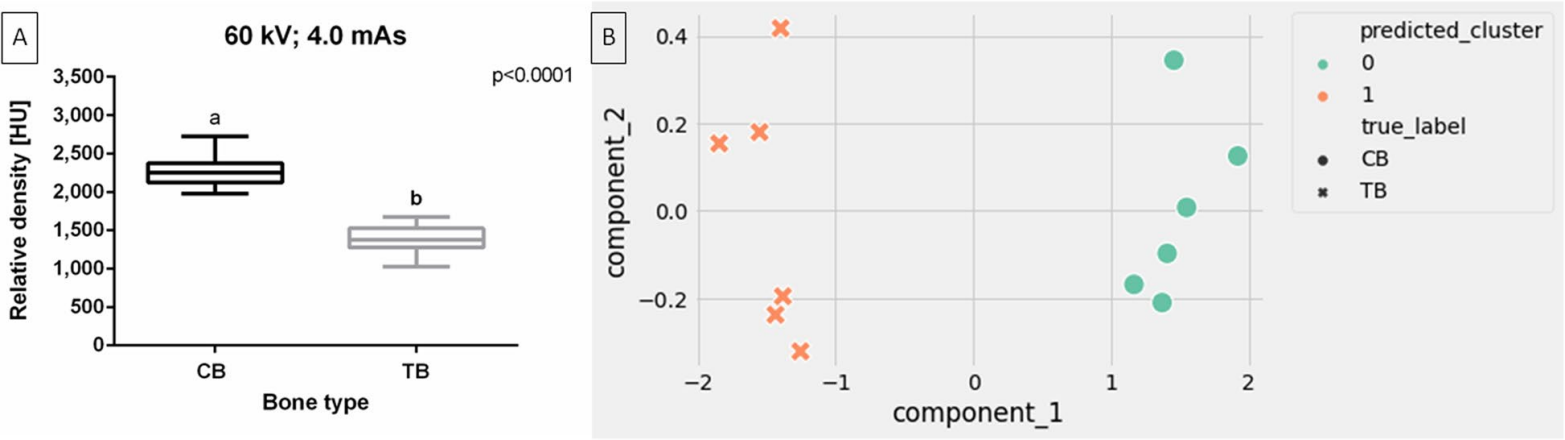
Composition of the relative bone mineral density (rBMD) [HU] of the cortical bone (CB) and trabecular bone (TB) of affected horses limbs. Comparison made for one studied X–ray tube setting (60 kV, 4,0 mAs). Data are represented by the median and range. Lowercase letters indicate differences between materials for *p* < 0.05 (**A**). Clustering of CB and TB of affected horses limbs. Clustering made for one studied X–ray tube setting (60 kV, 4,0 mAs) (**B**)

For the affected horses, the rBMD of the CB and TB differed between bone type (*p* < 0.0001). The rBMD was higher for CT than for TB (Fig. 10A) (*p* < 0.0001). The obtained values (*p* < 0.0001) for CB and TB allowed the clustering of bone types in affected limbs into two distinct classes (Fig. 10B).

The metrics of cluster cohesion and separation were high, with samples being very close to their respective clusters (SC closer to 1). When considering truth labels in the clustering evaluation, the similarity between true and predicted labels was full (ARI = 1). Therefore, the CDA parameters, including the Dur density standard and the 60 kV, 4.0 mAs X–ray tube setting, enabled radiographs with high CB and TB separation (Table 6).

**Table 6:** Clustering of the cortical bone (CB) and trabecular bone (TB) of healthy affected limbs. The Silhouette coefficient (SC), adjusted rand index (ARI), Intertia, and coordinates of cluster centers (x1, y1, x2, y2) measured for clustering of bone type for ten X–ray tube settings. Scores were ranked from best fit (SC closer to 1) to least fit (SB closer to -1) into its assigned cluster

Settings	SC	ARI	Intertia	× 1	y1	× 2	y2
60 kV, 4.0 mAs	0.852	1.00	1.166	1.479	0.0004	-1.479	-0.0004

## Discussion

One may observe that in healthy horse limbs, the rBMD of the long pastern bone differs between bone types, allowing the k–means clustering algorithm to effectively differentiate CB and TB from radiographs obtained using various X–ray tube settings. The k–means clustering exploratory statistical technique was used here to identifying groups by minimizing the sum of the squares of distances between data – represented by rBMD of CB and TB and the relative density of the density standard – and the corresponding centroid of the cluster [66]. Consequently, the identification of optimal CDA parameters involves considering the matching of both X–ray tube settings and density standards. The density standard paired with 60 kV and 4.0 mAs X–ray tube settings demonstrated the highest values in clustering metrics and, therefore, was selected and recommended for further research. Upon examining the detailed material characteristics and X–ray attenuation properties of all density standards, it becomes apparent, as discussed further, that even a small amount of copper in the aluminum alloy enhances the suitability of Dur as a density standard.

### Previously used CDA in equine bone evaluation

In the equine research, the density standard used in CDA method [39, 40, 61] is also referred to as radiographic bone aluminum equivalence (RBAE) [41, 48, 56, 57] or aluminum markers [50]. In all the studies mentioned, the density standard was either custom–made to order or crafted by the researchers themselves [39–41, 48, 50, 56, 57, 61]. None of the described density standards were commercially available, and to the best of our knowledge, there is currently no commercial complete system for CDA in horses available in the veterinary market. In the current study, all density standards were also custom–made by the authors. Each of the metals used, including the selected Dur, was commercially available. While machining required precision, it did not demand complex tools or high costs. Therefore, we anticipate that commercially available density standards and radiograph analysis software will soon be introduced to the equine veterinary market, extending the diagnostic capabilities of conventional radiography to include the CDA method.

Most authors do not specify the chemical composition of the density standard they use, simply noting that it is made of aluminum and has some level of gradation, such as 20 steps with 1 mm per step [41]. In one study, the density standard was described as aluminum markers with various thicknesses and uniform densities (type 6061 Al, 20.36 cm in length, 0.2–31.8 mm in thickness with a density of 2.70 g/cm^3^) [50]. In other studies, more detailed descriptions included the exact aluminum content (95.20–98,88 Mass% and 92.71–98.92 Atom% of aluminum) and specific dimensions (a volume of 3545.93 mm^3^ with a density of 2.65 g/cm^3^) [39, 40, 61]. Based on the studies to date, it can be concluded that aluminum density standards are widely acceptable for conventional radiography. However, there is no available literature investigating the use of density standards with different compositions in the CDA method in horses.

In the current study, the Alu density standard had a similar density and composition to the density standard described in other studies [39, 40, 50, 61], while the density of each subsequent density standard increased from Dur, through IrSi and IrNi to Cup. Metals with higher density, and necessarily different compositions, were chosen to increase the mass attenuation coefficient [60], and consequently the attenuation of the X–ray beam. It is worth noting that the aluminum alloy, such as Dur, rather than the previously used pure aluminum density standard, was considered optimal for quantifying the rBMD of the long pastern bone in the equine distal limb. In the case of projective images obtained through conventional radiography, mimicking the mass attenuation coefficient is often sufficient to produce an effective density standard. However, for 3D images obtained via CT, the linear attenuation coefficient must be considered [77]. When focusing on conventional radiography, which is commonly used to characterize radiographic signs of bone and teeth diseases, assess treatment outcome, and monitor healing processes [78], increasing the mass attenuation coefficient by incorporating filling materials or alloys with high and very high mass density is needed. Therefore, the proposed increase in the mass attenuation coefficient, corresponding with the increase in the X–ray beam attenuation, is considered beneficial.

The selection of a different material than those previously used [39, 40, 50, 61] may appear to be a result of the lack of consideration of metal properties in earlier studies [41, 48, 56, 57], or it may be related to differences in the bones under investigation. Recent equine CDA studies have mostly focused on the third metacarpal bone [41, 48, 50, 56, 57], and less frequently on the tarsometatarsal joint [40, 61], the third metatarsal bone [41], the femur [49], and teeth [39]. Therefore, further discussion regarding the effect of density standard composition on the results of scaled X–ray attenuation measurement is well justified.

### Density standard composition and X–ray beam settings

In most previous studies, bones and teeth were imaged using the CDA method with a single X–ray tube setting [39–41, 48, 56, 57, 61]. X–ray tube settings typically ranged from 60 to 70 kV and 1.2 to 10 mAs. Only Bowen et al. [50] explored a broader range of radiographic exposures, from 55 to 80 kV in 5 kV increments, at 15 mAs. Bowen et al. [50] reported that for each combination of intensity and exposure time, the BDI (the measure of relative density they used) of the aluminum marker (their density standard) showed a linear correlation with the BDI of bone. The authors concluded that BMD values derived by the CDA method were independent of exposure settings, which contradicts our findings. In the current study, the relative density values of Alu, Dur, IrSi, and IrNi decreased as exposure intensity and beam quantity increased, particularly at 90 kV and 4.0 mAs.

However, Bowen et al.’s [50] conclusions aligns with our results in that, across all X–ray tube settings, the rBMD of cortical bone remained higher than that of trabecular bone, allowing for differentiation between the two bone types. The discrepancies described may stem from differences in quantification methodology and the fact that Bowen et al. [50] did not take X–rays with the highest intensity beam (90 kV) due to the limitation of their X–ray machine, which ranged from 55 to 80 kV [50]. In the current study, the voltage ranged from 50 to 90 kV, with current and exposure time set at 1.2 mAs or 4.0 mAs, covering the typical X–ray tube settings used in equine veterinary radiography in clinical practice. A standard portable X–ray operates within a voltage range of 50 kV to 90 kV, adjusted in 5 or 10 kV steps. This range allows for obtaining high–quality radiographs of the third metacarpal bone (50 kV, 1.2 mAs) [48] as well as the cervical spine (90 kV, 4.0 mAs) [49, 79], making the CDA method feasible for most clinical applications of conventional radiography.

In the current study, the exact composition of each density standard was confirmed using SEM imaging. Since the precise chemical composition of the material can vary between individual samples, specific characterization is crucial for ensuring the reproducibility of X–ray beam attenuation properties. For example, the Dur standard density contained a higher Mass% of copper (17.53 Mass%) compared to the Alu standard density (3.56 Mass%), resulting in better differentiation of steps under high beam energy imaging (voltage settings of 80 and 90 kV and higher mAs settings). Conversely, the Cup standard density, with 56.24 Mass% copper, had too high a copper content to distinguish the steps at any X–ray tube settings. Given that the atomic mass of aluminum (26.982 u) is lower than that of copper (63.546 u), a higher proportion of copper shifts the mass attenuation coefficient, which is dependent on atomic mass, towards higher X–ray beam attenuation. Thus, further research is needed to identify aluminum–copper alloys which a copper content sufficient to differentiate all steps of the density standard, even at the highest X–ray tube settings.

The study also used two alternative iron–based density standards. Since the atomic mass of iron (55.845 u) is higher than that of aluminum but lower than cooper, the intermediate X–ray beam attenuation properties of the IrNi and IrSi density standards were expected and confirmed. However, their usefulness was limited due to the inability to distinguish steps in the density standard under low beam energy imaging (voltage settings of 50, 60, and 70 kV and both mAs settings). Among the two iron–based density standards, one iron alloy was doped nickel (58.693 u) and the other with silicon (28.085 u). Notable differences were observed in the slopes and intercepts of the X–ray beam attenuation line for the IrSi density standard compared to the IrNi density standard. Therefore, further studies are needed to identify iron–silicon alloys with a silicon content sufficient to allow differentiation of all steps of the density standard, even at the lowest X–ray tube settings.

### Clinical applications of CDA method in equine clinical practice

Dual–energy X–ray absorptiometry (DXA) is considered the most precise, accurate, and widely accepted technique for measuring bone mineral density (BMD) in human medicine [52–54]. However, in equine medicine, despite the availability of DXA [41, 42, 49–51], its use among equine practitioners is limited due to its high cost, time–consuming nature, and lack of portability [50]. The primary barriers to the widespread adoption of DXA in equine veterinary practice are its prohibitive cost, limited availability of human–specific systems adopted for horses, absence of equine–specific systems, and the requirement for general anesthesia during assessment [41, 50]. As a result, DXA availability is equine veterinary practice is restricted to few specialized veterinary research centers and academic clinics [49]. Studies using DXA in equines have demonstrated that BMD is a reliable predictor of bone strength, exercise–induced bone remodeling, and fracture risk [44, 45]. The CDA method also provides valuable data on skeleton development in young Thoroughbreds and helps identify predisposing factors for bone disorders [57]. CDA data are particularly relevant for breeding and training purposes, as pastured horses generally have greater BMD compared to stalled horses [48], and bone fractures in racehorses are often due to weakened bone areas or fatigue fractures that occur during training [41, 42]. Thus, while the CDA method offers similar clinical capabilities to DAX, its feasibility in field practice is potentially much greater.

Multi–detector computed tomography (MDCT) is a highly relevant method for assessing BMD in horses, particularly since MDCT can be performed under standing sedation [28, 30–34]. MDCT is clinically used to assess BMD in screening for diseases such as osteoporosis in humans [80], and it holds promise for monitoring bone weakness associated with overload or OA progression. However, while reducing the risks associated with general anesthesia, MDCT does not address the high costs of examination and requires transporting the horse to the clinic each time, which limits its widespread use for initial screening in field practice. Given that Yamada et al. [41] reported a strong positive correlated between BMD values obtained by the CDA method and those obtained by DAX and MDCT [41], we suggest that the CDA method could address the gap in rBMD assessment in field practice.

Equine practitioners routinely use conventional radiographs to assess bone health and diagnose abnormalities such as fractures and OA [50, 81]. Given that OA a leading cause of lameness and can end the sports career of equine athletes [82], enhancing the diagnostic capabilities of conventional radiography with the CDA method could significantly improve clinical practice by enabling more precise quantification and tracking of disease progression. In the current study, horses with distal limbs OA of varying severity showed significant differences in the rBMD of the long pastern bone between bone types, allowing the k–means clustering algorithm to effectively differentiate between CB and TB. With a high separation metric and full agreement between true and predicted labels, the optimal CDA protocol was identified and is recommended for further research. We believe that the proposed CDA protocol will serve as a foundation for developing commercially available equine–specific CDA systems, becoming a valuable tool for quantifying rBMD in the field practice.

### Limitations

It should be noted that BMD values can vary based on the measurement site [41] and between different bones [49]. Therefore, establishing landmarks is essential for achieving reproducible measurements, similar to the practices typically employed in human measurements [52, 53]. In the current study, landmarks were set halfway up the long pastern bone, parallel to the long axis. However, a comparative analysis across different equine bones – including the long pastern bone, third metacarpal bone, third metatarsal bone, and femur – is needed. Additionally, the quantification of BMD using conventional radiographs may vary depending on the conditions under which the images are obtained [50]. Vaccaro et al. [49] used Computer–Assisted Image Analysis Software (CAIAS) and noted that radiographic settings significantly influence the brightness and contrast of digital and digitized radiographs. However, the CAIAS method does not incorporate the use of a density standard, despite the fact that BMD is dependent on both material thickness and density [50]. Because conventional radiography provides two–dimensional images [9], factors such as beam angle, beam center positioning, X–ray tube–cassette distance, bone thickness and shape, and digital image processing must also be considered [50].

While BMD changes with age, the previously used CDA method has not been able to differentiate between the bones of growing and aged horses [41]. Additionally, no other age–related longitudinal studies have been conducted. In foals, a sex–related difference in BMD has been reported [16], but no follow–up studies have been carried out. The lack of specific BMD reference values for factors such as breed, sex, age, body weight, and usage limits the application of CDA analysis [49]. Therefore, in the pursuit of developing commercially available equine–specific CDA systems, future CDA–based studies should focus on establishing standardized imaging conditions and expected rBMD values for different groups of horses and bones, considering their thickness, shape, and any overload– or disease–related factors influencing equine BMD.

## Conclusions

Quantitative measurement of the relative bone mineral density (rBMD) of the equine long pastern bone using conventional radiography and metal–made density standards may have the potential to evaluate bone biology in field equine veterinary practice. In healthy horse limbs, the rBMD of long pastern bone differed between bone types, allowing the k–means clustering algorithm to differentiate cortical bone and trabecular bone from radiographs obtained using all X–ray tube settings.

The X–ray beam attenuation differs based on the atomic–mass–dependent properties of metals used to produce of the density standards. For the X–ray tube settings commonly used in veterinary radiography, the aluminum density standard absorbs too little X–ray radiation to be used in high–dose imaging. On the other hand, the cuprum standard absorbs too much X–ray radiation to be effective in this type of imaging. Increasing the proportion of copper in the aluminum alloy enhances the suitability of duralumin as a density standard; however, further increases and studies are needed to maintain its suitability at high voltages. Alternative iron–based alloys absorb too much X–ray radiation for low–dose imaging. However, increasing the proportion of silicon in the iron alloy may offer a potential direction for study in relation to the duralumin density standard. The duralumin density standard, combined with 60 kV and 4.0 mAs X–ray tube settings, exhibited the highest clustering metric values and was therefore considered optimal for further research.

In horses’ distal limbs affected by increasing severity of osteoarthritis, the rBMD of the long pastern bone differed between bone types, allowing the k–means clustering algorithm can differentiate cortical bone and trabecular bone in radiographs obtained using optimal computed digital absorptiometry parameters. Given the high separation metric and full agreement between true and predicted labels, the identified optimal computed digital absorptiometry parameters may be recommended for use in further research on the relative quantification of conventional radiographs and the examination of the distal limb in the field equine veterinary practice.

## Supplementary Information

Additional File 1. Pure aluminum (Alu) density standard. The X–ray beam attenuation measured using lines representing the lateral, middle, and medial aspect of density standard (A-E, U-Y) and the relative density [HU] versus distance [mm] charts (F-J, P-T) returned for following X–ray tube settings: 50 kV, 1.2 mAs (A, F); 60 kV, 1.2 mAs (B, G); 70 kV, 1.2 mAs (C, H); 80 kV, 1.2 mAs (D, I); 90 kV, 1.2 mAs (E, J); 50 kV, 4.0 mAs (P, U); 60 kV, 4.0 mAs (Q, V); 70 kV, 4.0 mAs (R, W); 80 kV, 4.0 mAs (S, X); and 90 kV, 4.0 mAS (T, Y). Linear regression charts and equations displayed for 1.2 mAs and 4.0 mAs data pairs for 50 kV (K), 60 kV (L), 70 kV (M), 80 kV (N), and 90 kV (O), respectively.

Additional File 2. The similarity between relative density under studied X–ray tube settings for pure aluminum (Alu) density standard summarized using the significance levels for slopes and intercepts. The similarity was tested using linear regressions and considered significant for *p* < 0.05. If the difference between slopes was not significant (*p* > 0.05), the difference between intercepts was tested. Additionally, the significant differences were marked with bold font.

Additional File 3. Aluminum alloy (duralumin, Dur) density standard. The X–ray beam attenuation measured using lines representing the lateral, middle, and medial aspect of density standard (A-E, U-Y) and the relative density [HU] versus distance [mm] charts (F-J, P-T) returned for following X–ray tube settings: 50 kV, 1.2 mAs (A, F); 60 kV, 1.2 mAs (B, G); 70 kV, 1.2 mAs (C, H); 80 kV, 1.2 mAs (D, I); 90 kV, 1.2 mAs (E, J); 50 kV, 4.0 mAs (P, U); 60 kV, 4.0 mAs (Q, V); 70 kV, 4.0 mAs (R, W); 80 kV, 4.0 mAs (S, X); and 90 kV, 4.0 mAS (T, Y). Linear regression charts and equations displayed for 1.2 mAs and 4.0 mAs data pairs for 50 kV (K), 60 kV (L), 70 kV (M), 80 kV (N), and 90 kV (O), respectively.

Additional File 4. The similarity between relative density under studied X–ray tube settings for aluminum alloy (duralumin, Dur) density standard summarized using the significance levels for slopes and intercepts. The similarity was tested using linear regressions and considered significant for *p* < 0.05. If the difference between slopes was not significant (*p* > 0.05), the difference between intercepts was tested. Additionally, the significant differences were marked with bold font.

Additional File 5. Cuprum alloy (Cup) density standard. The X–ray beam attenuation measured using lines representing the lateral, middle, and medial aspect of density standard (A-E, U-Y) and the relative density [HU] versus distance [mm] charts (F-J, P-T) returned for following X–ray tube settings: 50 kV, 1.2 mAs (A, F); 60 kV, 1.2 mAs (B, G); 70 kV, 1.2 mAs (C, H); 80 kV, 1.2 mAs (D, I); 90 kV, 1.2 mAs (E, J); 50 kV, 4.0 mAs (P, U); 60 kV, 4.0 mAs (Q, V); 70 kV, 4.0 mAs (R, W); 80 kV, 4.0 mAs (S, X); and 90 kV, 4.0 mAS (T, Y). Linear regression charts and equations displayed for 1.2 mAs and 4.0 mAs data pairs for 50 kV (K), 60 kV (L), 70 kV (M), 80 kV (N), and 90 kV (O), respectively.

Additional File 6. The similarity between relative density under studied X–ray tube settings for cuprum alloy (Cup) density standard summarized using the significance levels for slopes and intercepts. The similarity was tested using linear regressions and considered significant for *p* < 0.05. If the difference between slopes was not significant (*p* > 0.05), the difference between intercepts was tested. Additionally, the significant differences were marked with bold font.

Additional File 7. Iron-nickel alloy (IrNi) density standard. The X–ray beam attenuation measured using lines representing the lateral, middle, and medial aspect of density standard (A-E, U-Y) and the relative density [HU] versus distance [mm] charts (F-J, P-T) returned for following X–ray tube settings: 50 kV, 1.2 mAs (A, F); 60 kV, 1.2 mAs (B, G); 70 kV, 1.2 mAs (C, H); 80 kV, 1.2 mAs (D, I); 90 kV, 1.2 mAs (E, J); 50 kV, 4.0 mAs (P, U); 60 kV, 4.0 mAs (Q, V); 70 kV, 4.0 mAs (R, W); 80 kV, 4.0 mAs (S, X); and 90 kV, 4.0 mAS (T, Y). Linear regression charts and equations displayed for 1.2 mAs and 4.0 mAs data pairs for 50 kV (K), 60 kV (L), 70 kV (M), 80 kV (N), and 90 kV (O), respectively.

Additional File 8. The similarity between relative density under studied X–ray tube settings for iron-nickel alloy (IrNi) density standard summarized using the significance levels for slopes and intercepts. The similarity was tested using linear regressions and considered significant for *p* < 0.05. If the difference between slopes was not significant (*p* > 0.05), the difference between intercepts was tested. Additionally, the significant differences were marked with bold font.

Additional File 9. Iron-silicon alloy (IrSi) density standard. The X–ray beam attenuation measured using lines representing the lateral, middle, and medial aspect of density standard (A-E, U-Y) and the relative density [HU] versus distance [mm] charts (F-J, P-T) returned for following X–ray tube settings: 50 kV, 1.2 mAs (A, F); 60 kV, 1.2 mAs (B, G); 70 kV, 1.2 mAs (C, H); 80 kV, 1.2 mAs (D, I); 90 kV, 1.2 mAs (E, J); 50 kV, 4.0 mAs (P, U); 60 kV, 4.0 mAs (Q, V); 70 kV, 4.0 mAs (R, W); 80 kV, 4.0 mAs (S, X); and 90 kV, 4.0 mAS (T, Y). Linear regression charts and equations displayed for 1.2 mAs and 4.0 mAs data pairs for 50 kV (K), 60 kV (L), 70 kV (M), 80 kV (N), and 90 kV (O), respectively.

Additional File 10. The similarity between relative density under studied X–ray tube settings for iron-silicon alloy (IrSi) density standard summarized using the significance levels for slopes and intercepts. The similarity was tested using linear regressions and considered significant for *p*< 0.05. If the difference between slopes was not significant (*p* > 0.05), the difference between intercepts was tested. Additionally, the significant differences were marked with bold font.

## Data Availability

No datasets were generated or analysed during the current study.

## Abbreviations

AI: Artificial intelligence
Al: Aluminum
Alu: Pure aluminum
ARI: Adjusted rand index
Atom%: Atomic percentage
BDI: Brightness/darkness index
BMD: Bone mineral density
CAIAS: Computer–Assisted Image Analysis Software
CB: Cortical bone
CBCT: Cone bone computed tomography
CDA: Computed digital absorptiometry
Cl: Chlorine
CT: Computed tomography
Cu: Copper
Cup: Cuprum alloy
Dur: Duralumin
DXA: Dual–energy X–ray absorptiometry
Fe: Iron
HU: Hounsfield unit
IrNi: Iron–nickel alloy
IrSi: Iron–silicon alloy
Mass%: Mass percentage
MDCT: Multi–detector computed tomography
Mg: Magnesium
MIMICS: Materialises interactive medical image control system
ML: Machine learning
Mn: Manganese
MRI: Magnetic resonance imaging
Ni: Nickel
NP: Number of pixels
O: Oxygen
OA: Osteoarthritis
Pb: Lead
PB: Pixel brightness
PET: Positron emission tomography
RBAE: Radiographic bone aluminum equivalence
SC: Silhouette coefficients
SEM: Scanning electron microscope
Si: Silicon
TB: Trabecular bone
vBMD: Volumetric bone mineral density
Zn: Zinc
