# Supplementary material for: Selection of density standard and X–ray tube settings for computed digital absorptiometry in horses using the k–means clustering algorithm
Source: BMC Vet Res. 2025 Mar 13;21:165. doi: 10.1186/s12917-025-04591-5 (PMC11905476; doi:10.1186/s12917-025-04591-5)
Supplement: Supplementary file 1 — Additional File 1. Pure aluminum (Alu) density standard. The X–ray beam attenuation measured using lines representing the lateral, middle, and medial aspect of density standard (A-E, U-Y) and the relative density [HU] versus distance [mm] charts (F-J, P-T) returned for following X–ray tube settings: 50 kV, 1.2 mAs (A, F); 60 kV, 1.2 mAs (B, G); 70 kV, 1.2 mAs (C, H); 80 kV, 1.2 mAs (D, I); 90 kV, 1.2 mAs (E, J); 50 kV, 4.0 mAs (P, U); 60 kV, 4.0 mAs (Q, V); 70 kV, 4.0 mAs (R, W); 80 kV, 4.0 mAs (S, X); and 90 kV, 4.0 mAS (T, Y). Linear regression charts and equations displayed for 1.2 mAs and 4.0 mAs data pairs for 50 kV (K), 60 kV (L), 70 kV (M), 80 kV (N), and 90 kV (O), respectively. [file 12917_2025_4591_MOESM1_ESM.docx]

**Selection of density standard and X–ray tube settings for computed digital absorptiometry in horses using the k–means clustering algorithm**

Bernard Turek^1^, Marek Pawlikowski^2^, Krzysztof Jankowski^2^, Marta Borowska^3^, Katarzyna Skierbiszewska^1^, Tomasz Jasiński^1^, Małgorzata Domino^1,*^

^1^Department of Large Animal Diseases and Clinic, Institute of Veterinary Medicine, Warsaw University of Life Sciences (WULS – SGGW), Nowoursynowska 100, 02-797 Warszawa, Poland

^2^Institute of Mechanics and Printing, Warsaw University of Technology, Narbutta 85, 02-524 Warszawa, Poland

^3^Institute of Biomedical Engineering, Faculty of Mechanical Engineering, Białystok University of Technology, Wiejska 45C, 15-351 Bialystok, Poland

^*^Correspondence: [malgorzata_domino@sggw.edu.pl](mailto:malgorzata.domino@wp.pl)


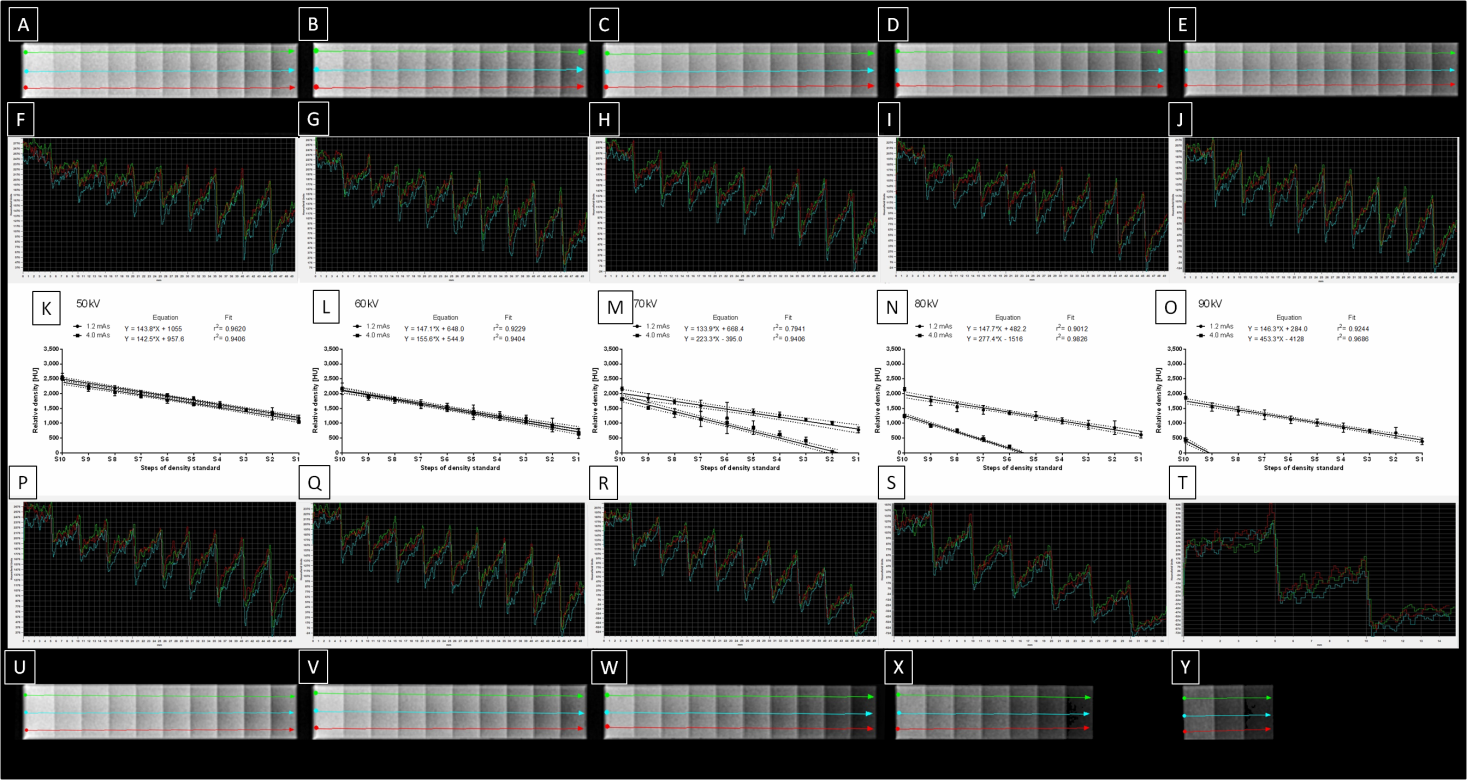


Additional File 1. Pure aluminum (Alu) density standard. The X–ray beam attenuation measured using lines representing the lateral, middle, and medial aspect of density standard (A-E, U-Y) and the relative density [HU] versus distance [mm] charts (F-J, P-T) returned for following X–ray tube settings: 50 kV, 1.2 mAs (A, F); 60 kV, 1.2 mAs (B, G); 70 kV, 1.2 mAs (C, H); 80 kV, 1.2 mAs (D, I); 90 kV, 1.2 mAs (E, J); 50 kV, 4.0 mAs (P, U); 60 kV, 4.0 mAs (Q, V); 70 kV, 4.0 mAs (R, W); 80 kV, 4.0 mAs (S, X); and 90 kV, 4.0 mAS (T, Y). Linear regression charts and equations displayed for 1.2 mAs and 4.0 mAs data pairs for 50 kV (K), 60 kV (L), 70 kV (M), 80 kV (N), and 90 kV (O), respectively.
