# Supplementary material for: Selection of density standard and X–ray tube settings for computed digital absorptiometry in horses using the k–means clustering algorithm
Source: BMC Vet Res. 2025 Mar 13;21:165. doi: 10.1186/s12917-025-04591-5 (PMC11905476; doi:10.1186/s12917-025-04591-5)
Supplement: Supplementary file 4 — Additional File 4. The similarity between relative density under studied X–ray tube settings for aluminum alloy (duralumin, Dur) density standard summarized using the significance levels for slopes and intercepts. The similarity was tested using linear regressions and considered significant for p < 0.05. If the difference between slopes was not significant (p > 0.05), the difference between intercepts was tested. Additionally, the significant differences were marked with bold font. [file 12917_2025_4591_MOESM4_ESM.docx]

Additional File 4**.** The similarity between relative density under studied X–ray tube settings for aluminum alloy (duralumin, Dur) density standard summarized using the significance levels for slopes and intercepts. The similarity was tested using linear regressions and considered significant for p < 0.05. If the difference between slopes was not significant (p > 0.05), the difference between intercepts was tested. Additionally, the significant differences were marked with bold font.

| **Settings** | **Equation** | **60 kV; 1.2 mAs** | **70 kV; 1.2 mAs** | **80 kV; 1.2 mAs** | **90 kV; 1.2 mAs** | **50 kV; 4.0 mAs** | **60 kV; 4.0 mAs** | **70 kV; 4.0 mAs** | **80 kV; 4.0 mAs** | **90 kV; 4.0 mAs** |
| --- | --- | --- | --- | --- | --- | --- | --- | --- | --- | --- |
| **50 kV; 1.2 mAs** | slope | p=0.119 | p=0.609 | p=0.548 | p=0.100 | p=0.605 | **p=0.031** | **p<0.0001** | **p<0.0001** | **p<0.0001** |
|  | intercept | **p<0.0001** | **p<0.0001** | **p<0.0001** | **p<0.0001** | **p<0.0001** |  |  |  |  |
| **60 kV; 1.2 mAs** | slope |  | p=0.457 | p=0.387 | p=0.886 | p=0.054 | p=0.563 | **p<0.0001** | **p<0.0001** | **p<0.0001** |
|  | intercept |  | p=0.349 | **p<0.0001** | **p<0.0001** | **p<0.0001** | p=0.852 |  |  |  |
| **70 kV; 1.2 mAs** | slope |  |  | p=0.995 | p=0.396 | p=0.379 | p=0.217 | **p<0.0001** | **p<0.0001** | **p<0.0001** |
|  | intercept |  |  | **p<0.0001** | **p<0.0001** | **p<0.0001** | p=0.279 |  |  |  |
| **80 kV; 1.2 mAs** | slope |  |  |  | p=0.323 | p=0.303 | p=0.154 | **p<0.0001** | **p<0.0001** | **p<0.0001** |
|  | intercept |  |  |  | **p=0.0009** | **p<0.0001** | **p<0.0001** |  |  |  |
| **90 kV; 1.2 mAs** | slope |  |  |  |  | **p=0.046** | p=0.677 | **p<0.0001** | **p<0.0001** | **p<0.0001** |
|  | intercept |  |  |  |  |  | **p<0.0001** |  |  |  |
| **50 kV; 4.0 mAs** | slope |  |  |  |  |  | **p=0.013** | **p<0.0001** | **p<0.0001** | **p<0.0001** |
|  | intercept |  |  |  |  |  |  |  |  |  |
| **60 kV; 4.0 mAs** | slope |  |  |  |  |  |  | **p<0.0001** | **p<0.0001** | **p<0.0001** |
|  | intercept |  |  |  |  |  |  |  |  |  |
| **70 kV; 4.0 mAs** | slope |  |  |  |  |  |  |  | **p=0.014** | **p=0.006** |
|  | intercept |  |  |  |  |  |  |  |  |  |
| **80 kV; 4.0 mAs** | slope |  |  |  |  |  |  |  |  | **p=0.023** |
|  | intercept |  |  |  |  |  |  |  |  |  |
