# Supplementary material for: Selection of density standard and X–ray tube settings for computed digital absorptiometry in horses using the k–means clustering algorithm
Source: BMC Vet Res. 2025 Mar 13;21:165. doi: 10.1186/s12917-025-04591-5 (PMC11905476; doi:10.1186/s12917-025-04591-5)
Supplement: Supplementary file 5 — Additional File 5. Cuprum alloy (Cup) density standard. The X–ray beam attenuation measured using lines representing the lateral, middle, and medial aspect of density standard (A-E, U-Y) and the relative density [HU] versus distance [mm] charts (F-J, P-T) returned for following X–ray tube settings: 50 kV, 1.2 mAs (A, F); 60 kV, 1.2 mAs (B, G); 70 kV, 1.2 mAs (C, H); 80 kV, 1.2 mAs (D, I); 90 kV, 1.2 mAs (E, J); 50 kV, 4.0 mAs (P, U); 60 kV, 4.0 mAs (Q, V); 70 kV, 4.0 mAs (R, W); 80 kV, 4.0 mAs (S, X); and 90 kV, 4.0 mAS (T, Y). Linear regression charts and equations displayed for 1.2 mAs and 4.0 mAs data pairs for 50 kV (K), 60 kV (L), 70 kV (M), 80 kV (N), and 90 kV (O), respectively. [file 12917_2025_4591_MOESM5_ESM.docx]

**
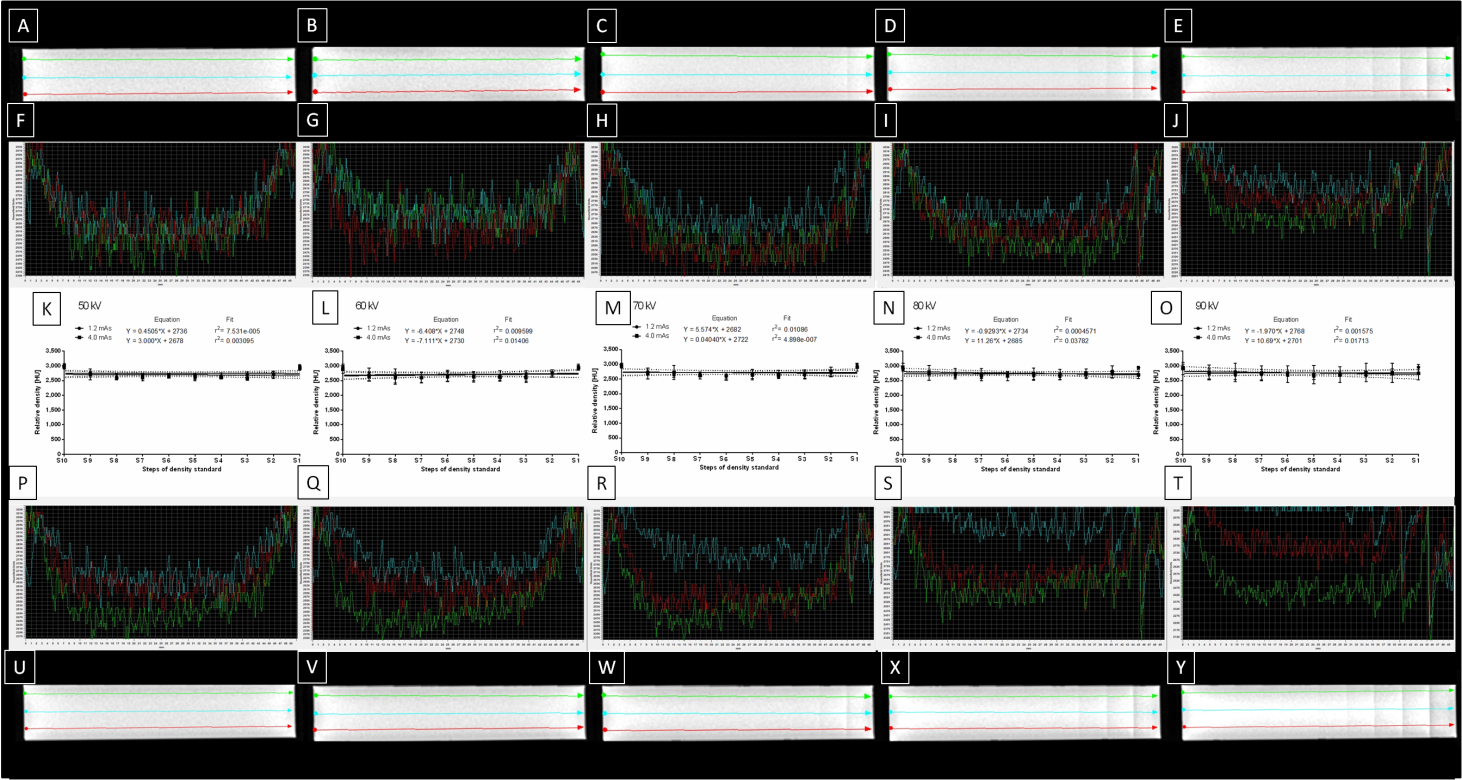
**

Additional File 5. Cuprum alloy (Cup) density standard. The X–ray beam attenuation measured using lines representing the lateral, middle, and medial aspect of density standard (A-E, U-Y) and the relative density [HU] versus distance [mm] charts (F-J, P-T) returned for following X–ray tube settings: 50 kV, 1.2 mAs (A, F); 60 kV, 1.2 mAs (B, G); 70 kV, 1.2 mAs (C, H); 80 kV, 1.2 mAs (D, I); 90 kV, 1.2 mAs (E, J); 50 kV, 4.0 mAs (P, U); 60 kV, 4.0 mAs (Q, V); 70 kV, 4.0 mAs (R, W); 80 kV, 4.0 mAs (S, X); and 90 kV, 4.0 mAS (T, Y). Linear regression charts and equations displayed for 1.2 mAs and 4.0 mAs data pairs for 50 kV (K), 60 kV (L), 70 kV (M), 80 kV (N), and 90 kV (O), respectively.
