# Supplementary material for: Selection of density standard and X–ray tube settings for computed digital absorptiometry in horses using the k–means clustering algorithm
Source: BMC Vet Res. 2025 Mar 13;21:165. doi: 10.1186/s12917-025-04591-5 (PMC11905476; doi:10.1186/s12917-025-04591-5)
Supplement: Supplementary file 8 — Additional File 8. The similarity between relative density under studied X–ray tube settings for iron-nickel alloy (IrNi) density standard summarized using the significance levels for slopes and intercepts. The similarity was tested using linear regressions and considered significant for p < 0.05. If the difference between slopes was not significant (p > 0.05), the difference between intercepts was tested. Additionally, the significant differences were marked with bold font. [file 12917_2025_4591_MOESM8_ESM.docx]

Additional File 8**.** The similarity between relative density under studied X–ray tube settings for iron-nickel alloy (IrNi) density standard summarized using the significance levels for slopes and intercepts. The similarity was tested using linear regressions and considered significant for p < 0.05. If the difference between slopes was not significant (p > 0.05), the difference between intercepts was tested. Additionally, the significant differences were marked with bold font.

| **Settings** | **Equation** | **60 kV; 1.2 mAs** | **70 kV; 1.2 mAs** | **80 kV; 1.2 mAs** | **90 kV; 1.2 mAs** | **50 kV; 4.0 mAs** | **60 kV; 4.0 mAs** | **70 kV; 4.0 mAs** | **80 kV; 4.0 mAs** | **90 kV; 4.0 mAs** |
| --- | --- | --- | --- | --- | --- | --- | --- | --- | --- | --- |
| **50 kV; 1.2 mAs** | slope | p=0.873 | p=0.587 | p=0.388 | **p=0.020** | p=0.648 | p=0.789 | p=0.872 | **p=0.009** | **p<0.0001** |
|  | intercept | p=0.939 | p=0.807 | p=0.370 |  | p=0.597 | p=0.294 | p=0.948 |  |  |
| **60 kV; 1.2 mAs** | slope |  | p=0.746 | p=0.545 | **p=0.049** | p=0.555 | p=0.922 | p=0.754 | **p=0.023** | **p=0.0004** |
|  | intercept |  | p=0.757 | p=0.465 |  | p=0.647 | p=0.359 | p=0.890 |  |  |
| **70 kV; 1.2 mAs** | slope |  |  | p=0.732 | **p=0.032** | p=0.281 | p=0.828 | p=0.459 | **p=0.014** | **p<0.0001** |
|  | intercept |  |  | p=0.157 |  | p=0.398 | p=0.161 | p=0.861 |  |  |
| **80 kV; 1.2 mAs** | slope |  |  |  | **p=0.040** | p=0.148 | p=0.620 | p=0.279 | **p=0.017** | **p<0.0001** |
|  | intercept |  |  |  |  | p=0.783 | p=0.682 | p=0.320 |  |  |
| **90 kV; 1.2 mAs** | slope |  |  |  |  | **p=0.003** | p=0.056 | **p=0.010** | p=0.520 | **p=0.014** |
|  | intercept |  |  |  |  |  | p=0.149 |  | p=0.923 |  |
| **50 kV; 4.0 mAs** | slope |  |  |  |  |  | p=0.478 | p=0.764 | **p=0.002** | **p<0.0001** |
|  | intercept |  |  |  |  |  | p=0.573 | p=0.545 |  |  |
| **60 kV; 4.0 mAs** | slope |  |  |  |  |  |  | p=0.670 | **p=0.025** | **p=0.0004** |
|  | intercept |  |  |  |  |  |  | p=0.260 |  |  |
| **70 kV; 4.0 mAs** | slope |  |  |  |  |  |  |  | **p=0.004** | **p<0.0001** |
|  | intercept |  |  |  |  |  |  |  |  |  |
| **80 kV; 4.0 mAs** | slope |  |  |  |  |  |  |  |  | p=0.094 |
|  | intercept |  |  |  |  |  |  |  |  | p=0.643 |
