# Supplementary material for: Selection of density standard and X–ray tube settings for computed digital absorptiometry in horses using the k–means clustering algorithm
Source: BMC Vet Res. 2025 Mar 13;21:165. doi: 10.1186/s12917-025-04591-5 (PMC11905476; doi:10.1186/s12917-025-04591-5)
Supplement: Supplementary file 10 — Additional File 10. The similarity between relative density under studied X–ray tube settings for iron-silicon alloy (IrSi) density standard summarized using the significance levels for slopes and intercepts. The similarity was tested using linear regressions and considered significant for p< 0.05. If the difference between slopes was not significant (p > 0.05), the difference between intercepts was tested. Additionally, the significant differences were marked with bold font. [file 12917_2025_4591_MOESM10_ESM.docx]

Additional File 10**.** The similarity between relative density under studied X–ray tube settings for iron-silicon alloy (IrSi) density standard summarized using the significance levels for slopes and intercepts. The similarity was tested using linear regressions and considered significant for p < 0.05. If the difference between slopes was not significant (p > 0.05), the difference between intercepts was tested. Additionally, the significant differences were marked with bold font.

| **Settings** | **Equation** | **60 kV; 1.2 mAs** | **70 kV; 1.2 mAs** | **80 kV; 1.2 mAs** | **90 kV; 1.2 mAs** | **50 kV; 4.0 mAs** | **60 kV; 4.0 mAs** | **70 kV; 4.0 mAs** | **80 kV; 4.0 mAs** | **90 kV; 4.0 mAs** |
| --- | --- | --- | --- | --- | --- | --- | --- | --- | --- | --- |
| **50 kV; 1.2 mAs** | slope | p=0.689 | p=0.249 | p=0.078 | **p=0.010** | p=0.911 | p=0.675 | p=0.612 | **p=0.0001** | **p=0.0002** |
|  | intercept | p=0.284 | p=0.598 | **p=0.040** |  | p=0.206 | **p=0.036** | **p=0.037** |  |  |
| **60 kV; 1.2 mAs** | slope |  | p=0.516 | p=0.231 | **p=0.047** | p=0.645 | p=0.960 | p=0.922 | **p=0.001** | **p=0.0009** |
|  | intercept |  | p=0.513 | p=0.440 |  | p=0.797 | p=0.277 | p=0.328 |  |  |
| **70 kV; 1.2 mAs** | slope |  |  | p=0.525 | p=0.108 | p=0.268 | p=0.593 | p=0.584 | **p=0.002** | **p=0.002** |
|  | intercept |  |  | p=0.084 | **p=0.015** | p=0.378 | p=0.074 | p=0.079 |  |  |
| **80 kV; 1.2 mAs** | slope |  |  |  | p=0.300 | p=0.106 | p=0.305 | p=0.270 | **p=0.006** | **p=0.005** |
|  | intercept |  |  |  | p=0.398 | p=0.679 | p=0.591 | p=0.720 |  |  |
| **90 kV; 1.2 mAs** | slope |  |  |  |  | **p=0.020** | p=0.080 | p=0.056 | p=0.061 | **p=0.026** |
|  | intercept |  |  |  |  |  | p=0.940 | p=0.749 | p=0.860 |  |
| **50 kV; 4.0 mAs** | slope |  |  |  |  |  | p=0.633 | p=0.578 | **p=0.0004** | **p=0.0004** |
|  | intercept |  |  |  |  |  | p=0.430 | p=0.511 |  |  |
| **60 kV; 4.0 mAs** | slope |  |  |  |  |  |  | p=0.969 | **p=0.003** | **p=0.002** |
|  | intercept |  |  |  |  |  |  | p=0.846 |  |  |
| **70 kV; 4.0 mAs** | slope |  |  |  |  |  |  |  | **p=0.001** | **p=0.001** |
|  | intercept |  |  |  |  |  |  |  |  |  |
| **80 kV; 4.0 mAs** | slope |  |  |  |  |  |  |  |  | p=0.385 |
|  | intercept |  |  |  |  |  |  |  |  | p=0.420 |
